# Experimental and Numerical Study on Mechanical Properties of Threaded-Sleeve-Type Disconnectable Coupling for Foundation Pit Engineering

**DOI:** 10.3390/ma19214682

**Published:** 2026-11-03

**Authors:** Zhitian Xie, Bingzhi Li, Yong Fu, Wei Song, Jiahui Liao, Xiaokai Niu

**Affiliations:** 1 Beijing Municipal Engineering Research Institute, Beijing 100037, China; mlfgnr@163.com (Z.X.); 25115180@bjtu.edu.cn (B.L.); songwei170@163.com (W.S.); liao1319@126.com (J.L.); 2 Beijing Construction Engineering Group Co., Ltd., Beijing 100055, China; wh3100206660@163.com

**Keywords:** foundation pit engineering, disconnectable coupling, mechanical properties, prototype test, numerical simulation

## Abstract

To overcome the performance shortcomings of disconnectable couplings in foundation pit support systems, a threaded-sleeve-type disconnectable coupling was designed and developed in this study. An axial loading test was performed to investigate the bearing capacity of the coupling. Its yield load, initial compression stiffness, load–displacement curve, stress distribution, and failure mode were determined. A finite element analysis parametric study was performed and formulae for calculating the yield load and compressive stiffness proposed. The following conclusions were drawn from the results: (1) The coupling has good bearing performance. The yield load is 10,509.96 kN, the ultimate load is 12,980.2 kN, and the initial compression stiffness is 4215.17 kN·mm^−1^. The failure of the coupling is characterized by an outward bulging of the nut part and shearing of the thread. (2) The coupling has good ductility. Strain concentration occurs at the screw. (3) The bearing capacity of the coupling is positively correlated with the outer diameter of the nut, nut thickness, and screw thickness and linearly inversely related to the elongation and screw pitch. (4) The established calculation formulae can accurately estimate the yield load and initial compression stiffness of the coupling.

## 1. Introduction

Steel support systems are commonly used in foundation pit engineering. The support system resists earth pressure and ensures stability of the foundation pit by establishing a support structure inside the foundation pit. Such systems are widely used in foundation pit engineering because of their light weight, easy hoisting, fast assembly, convenient disassembly and reuse, and active prestressing. However, steel supports have not yet reached their expected bearing capacities in practical engineering applications. This is because current steel wedge-type disconnectable couplings results in a local weakening of the internal support system, which prevents the steel support from reaching its full bearing performance and is an important cause of foundation pit accidents [1,2].

A disconnectable coupling is a node set at the end of a steel support to connect the retaining structure to the steel support. It allows for the length of the steel support to be adjusted and applies a pre-axial force [3,4], which can suppress excessive deformation of the retaining structure [5]. Currently, steel wedge-type disconnectable couplings are widely used in subway foundation pits in China owing to their convenient installation. However, defects in such couplings are also very prominent. The complete separation between the steel wedge and active end results in poor structural integrity [6]. The force is primarily concentrated at the steel wedge, which has a force area smaller than that of the steel support [7,8]. This makes the disconnectable coupling a weak node in the steel support system, as shown in Figure 1. At the same time, each disconnectable coupling is equipped with multiple steel wedges. The steel wedge often experiences a point force and cannot be fully wedged during the combination process [9]. This further reduces the performance of the coupling. Insufficient bearing capacity in a disconnectable coupling can easily lead to the destruction of the entire structural system [10,11,12,13]. Fang [14] found that installing more nodes results in poor overall stiffness of the steel support. This makes the nodes easily deformed and sometimes even damaged when the node structure is poorly designed or when construction does not meet design requirements. The high risk of potential damage to the foundation pit needs to be mitigated through rational design, strict on-site management, and improved construction technology. Zhang et al. [15,16] compared and analyzed the experimental results for a steel wedge disconnectable coupling in a steel support with the theoretical calculation results. They found that the bearing capacity of the coupling is equivalent to only 50% of a 18 m steel support and its initial compression stiffness 78% of the steel support. On 15 November 2008, the foundation pit of the Xianghu Station on Hangzhou Metro Line 1 suffered a collapse, as shown in Figure 2. Zhang [17] pointed out that one cause of the accident is the weak link of the support system. If steel trusses or pipes have been used for internal support, a sample of the joint connection structure should be provided for further investigations. On 14 July 2010, a steel support fell off during the construction of the foundation pit in the open-cut station at Shunyi Station on Beijing Metro Line 15, as shown in Figure 3. The main cause of the accident was the loosening of the steel wedge at its disconnectable coupling.

Studies have been conducted to address the shortcomings of steel wedge active disconnectable couplings. Zhang et al. [18] proposed a hoop-type disconnectable coupling in which the envelope structure is supported by the active end through friction. However, the specimen failed immediately when the pressure exceeded the maximum static friction in indoor axial compression tests. The frictional active end must, therefore, be improved to ensure safety. Huang et al. [19] proposed an active disconnectable coupling with bolt fastening for axial force locking and performed field tests to verify its pre-axial force retention. However, the requirement to ensure that the bolts on both sides are fastened to the same degree makes the coupling difficult to erect. Zhang et al. [20,21,22,23] proposed a bolt-fastening tapered-wedge disconnectable coupling and performed indoor loading and field tests. Although the coupling exhibited strong pre-axial force retention, its bearing performance was not significantly improved compared with that of traditional single-box and double-box active ends. To this end, it was further proposed that the fixed end of the mold bag concrete [24,25] should be designed to allow for modular construction of the end node in the internal support system and alleviate the eccentricity effect. Jia et al. [26] investigated servo steel support technology and verified its safety, reliability, and ability to suppress deformation through field measurements and numerical analyses. The study provided a reference for similar engineering designs. Wang et al. [27] developed an innovative servo–concrete support system. The flexural performance of the wall was improved through real-time adjustments of the axial force in the steel support by the servo system. Field tests were conducted during the excavation of a foundation pit. Xu et al. [28] developed an assembled H-shaped steel support (AHSS) with a servo system. They analyzed the axial force in the struts and performed on-site monitoring of the horizontal displacement in the retaining wall, the surface settlement, and the displacement of the adjacent tunnel. The analysis results show that the servo system can provide a stable axial force for each AHSS. However, this type of disconnectable coupling has high costs and operates in a harsh environment. Although numerous new active terminals and measures have been proposed to date, not many of them are available for practical engineering applications, and there are few theoretical methods for calculating their bearing capacities. It is, therefore, necessary to develop a disconnectable coupling with strong bearing performance, simple structural design, and convenient operation and to perform a series of studies on the developed coupling.

In consideration of the above, a threaded-sleeve-type disconnectable coupling with high integrity, strong bearing performance, simple structural design, and convenient practical operation was designed and developed in this study. The bearing performance of the coupling under an axial load was evaluated through full-scale tests, and its strain distribution, yield load, ultimate load, initial compression stiffness, and failure characteristics were determined. Further parametric analyses were performed using the finite element method. Finally, modified calculation formulae were proposed for the yield load and initial compression stiffness of the coupling.

## 2. Axial Load Test of Threaded-Sleeve-Type Disconnectable Coupling

### 2.1. Overview of Coupling

The main components of the threaded-sleeve-type disconnectable coupling are a screw part, nut, and fixed part. The nut part is set on the threaded screw of the screw part and can be rotated to any position on the screw. The contact surface between the nut and fixed parts is connected using bolts. The screw part is nested in but not connected to the fixed part, which guides their relative sliding. The erection process is shown in Figure 4. The coupling is fabricated from Q235b steel and has a thread with a coarse thread pitch of 8 mm. The detailed structural specifications are shown in Figure 5.

### 2.2. Loading Device and Method

A multifunctional joint loading structure test system was used for loading, as shown in Figure 6. This test is mainly intended to reveal the typical failure mechanism and load–displacement characteristics.

The specimen was loaded over two stages comprising the preloading and formal loading stages. Preloading was performed prior to formal loading to eliminate the influence of cracks in the test results. During preloading, the load was increased from 0 to 800 kN at a rate of 0.5 mm/min before unloading to 0 N. This preloading aims to close assembly gaps of thread teeth and end-plate interfaces, stabilize surface contact conditions, remove assembly looseness of the steel coupling, and verify the normal operation of force and displacement measuring instruments. The preload of 800 kN shall be determined according to the expected working preload and designed axial force of the joint. In the formal loading stage, the sample was uniformly loaded at a rate of 0.5 mm/min until the bearing capacity of the specimen no longer increased or the specimen incurred obvious damage—the vertical displacement transducer exceeded 50 mm.

### 2.3. Measuring Point Arrangement

The arrangement of the strain measurement points on the specimen is shown in Figure 7. Strain gauges were arranged on the N and W sides with 10 measuring points on each side. Three groups of sensors, denoted as DMAS1 through DMAS3, were configured for displacement acquisition, as shown in Figure 8. DMAS1 measured the horizontal deformation in the middle of the nut part, while DMAS2 and DMAS3 measured the vertical displacement of the specimen. The average values of the data collected by DMAS2 and DMAS3 were used for the load in the specimen load–displacement curve, which corresponds to the central axis of the specimen.

## 3. Test Results

### 3.1. Failure Mode

When the specimen was loaded to 10,509.96 kN, the nut part of the specimen exhibited slight buckling, as shown in Figure 9. When the load was increased to 12,980 kN, the nut part became more evident, as shown in Figure 10. Loading was stopped when the specimen exhibited a large deformation. To further investigate the failure mode of the thread-casing-type active winding end, the nut part was cut along the vertical direction. Figure 11 shows that shear failure occurred at the thread, with its first five rings exhibiting the most significant damage.

### 3.2. Analysis of Test Data

#### 3.2.1. Load–Strain Curve

The load–strain curves of the specimen are shown in Figure 12. Generally, similar load–strain responses are observed between the N-side and W-side measuring points, verifying the overall stability of the threaded-sleeve-type disconnectable coupling under axial compression. However, evident asymmetric deformation exists for counterpart sensors, especially for #6, #7 and #8, with prominent strain development. Figure 12a,b illustrate the load–strain curves of the N-side and W-side, respectively. Abrupt strain growth, representing the onset of nonlinear deformation rather than fully developed plastic strain, occurs at N6/W6, N7/W7 and N8/W8 when the load reaches approximately 10,509 kN. At this stage, the strain concentrates mainly in the middle-lower nut section and the upper fixed-part section. As the load further rises, strains at these positions increase rapidly.

To provide a more intuitive comparison of the load–strain trends on the N-side and W-side of the disconnectable coupling, Figure 13 compares the load–strain curves for N6/W6, N7/W7 and N8/W8. The point-to-point comparison shows that N6 bears larger compressive strain with milder post-peak degradation relative to W6, while W6 exhibits early stiffness reduction. W7 fails at 11,939 kN, with faster stiffness deterioration, which is much earlier than N7. N8 maintains a stable bearing capacity with slow stiffness loss, whereas W8 suffers rapid post-peak load decay. Subsequently, measuring points N6/W6, N7/W7 and N8/W8 fail at 12,980 kN. The final compressive strains are −1.339% (N6), −1.537% (W6), −1.602% (N7), −1.709% (W7), −1.693% (N8) and −1.307% (W8). Deformation is mainly localized in the middle of the nut, consistent with the test failure mode of bulging-thread shearing on the nut. Since the threaded-sleeve joint is a high-strength mechanical connector, the assembly behaves like an axially loaded steel tubular member, leading to strain concentration in the nut mid-section.

#### 3.2.2. Load–Displacement Curve

Figure 14 shows the load–displacement curve of the specimen along the vertical direction. The curve is linear at the initial stage of loading, and the specimen exhibited good strength and stiffness. When the load reached 10,509.96 kN, the specimen entered the yield stage and exhibited an evident yield plateau. Plastic deformation of the specimen began to occur, and the slope of the curve gradually increased. When the load reached 12,980.2 kN, the specimen entered the necking stage. This proves that the movable end has good ductility and the potential to improve construction safety. The bearing performance indices of the threaded-sleeve-type active winding end were obtained by analyzing the load–displacement curve. The load corresponding to 0.2% residual deformation is defined as the yield load (*F_y,Ex_*), and the average slope of the curve from the origin to the yield load (*F_y,Ex_*) is defined as the initial compressive stiffness (*k*_Ex_) of the specimen [29]. Accordingly, the yield load is 10,509.96 kN, the ultimate load is 12,980.2 kN, and the initial compressive stiffness (*k*_Ex_) of the specimen, which is defined as the average slope of the curve from the origin to the yield load, is 4215.17 kN · mm^−1^.

Figure 15 shows the load–displacement curve for the entire process along the horizontal direction of the specimen. The steps in the curve are due to the shear failure of the thread. When one layer of thread was sheared, horizontal displacement of the nut part of the specimen occurred. The stepped curve decreases in a layer-by-layer manner because the thread was more likely to be damaged when the steel reached its yield point. The five obvious plateaus in the curve are consistent with the destruction of five layers of threads in the specimen, as described in Section 2.1.

## 4. Numerical Simulations

Threaded-sleeve-type disconnectable couplings are composed of multiple components with diverse structural forms and parameters. Because changes in the parameters can affect the bearing performance of the threaded-sleeve-type active end, a numerical model of the active end was established by Abaqus 2022 software to investigate their effects. The load–displacement curves and failure modes obtained through numerical analysis are compared with the experimental results to verify the accuracy of the finite element model.

### 4.1. Establishment of Finite Element Model (FEM)

An equal-size numerical model was established based on the dimensions of the specimen used in the test. The numerical model was divided into 75,682 grid points, as shown in Figure 16a. Solid hexahedral elements are employed for all steel parts. A mesh-convergence study was conducted on the thread-engagement zone prone to stress-concentration and shear-dominated failure. Three thread-tooth mesh sizes (10 mm, 5 mm, and 1 mm) were tested with unchanged non-thread meshes, using ultimate bearing capacity, maximum thread-tooth equivalent stress and computation time as metrics. Less than 2.3% and 3.1% discrepancies in ultimate capacity and peak equivalent stress existed between medium and fine meshes. Balancing accuracy and cost, the 5 mm medium mesh was adopted for parametric analyses.

The three-line constitutive model proposed by Bahaari et al. [30] was used in this study. The key material constants for this constitutive model are as follows: Young’s modulus (E = 206 GPa), Poisson’s ratio (*v* = 0.3), yield strength and ultimate strength are determined according to References [25,31]. To simulate the axial loading behavior in the test accurately, a reference point RP-1 was set at the center point of the endplate outer surface and motion coupling was established with the endplate outer surface. Force-controlled axial loading was applied at RP-1 to simulate the restraint effect of the loading plate in the test setup. The load was applied as shown in Figure 16b. The boundary condition of the test piece is fully fixed at the bottom.

Surface-to-surface penalty contact with finite sliding is employed for thread-to-thread and end-plate interfaces. Following steel-structure specifications, the steel-to-steel friction coefficient is set to 0.35. Real thread geometries are built, with separate contact pairs defined for each engaged thread surface to simulate thread extrusion and shear responses. Both force and displacement convergence criteria are enabled for nonlinear contact iterations: the residual force tolerance is 0.5% of the reference applied force, and the displacement correction tolerance is 1% to ensure convergence of strongly-nonlinear contact analysis.

### 4.2. Comparison of Numerical and Experimental Results

#### 4.2.1. Failure Mode

Figure 17 shows a comparison between the numerical simulation and experimental results for the specimen. The numerical simulation results for the compression at the extended part of the screw and the stress at the thread are consistent with the experimental results. The stress is mainly concentrated at the uppermost thread of the contact between the screw and nut parts, whereas the stress in the other parts is small and the deformation is not obvious.

#### 4.2.2. Load–Displacement Curve

Because a continuum medium model is adopted in the finite element software, only the load–displacement curve before specimen shear failure is analyzed in this section. Figure 18 shows a comparison between the load–displacement curve obtained using the finite element calculation and the experimental skeleton curve. The finite element curve is consistent with the experimental test curve in the elastic stage and in good agreement in the elastic–plastic and plastic stages. The coefficient of determination, R^2^, reaches 0.972 for the load–displacement datasets, and the relative error of the characteristic yield load is within 4.2%, demonstrating a favorable prediction accuracy of the numerical model. The stress in the specimen was, therefore, calculated accurately in the numerical analysis.

The established finite element model of the threaded-sleeve-type disconnectable coupling can not only accurately predict the deformation trend of the specimen and the failure state and detailed stress characteristics of its components but also the load–displacement curve. It should be noted that the continuum finite-element model in this study can simulate the elastoplastic behavior, stress distribution and contact state only before thread shear failure initiates, and cannot reproduce the full post-peak progressive shearing-off of thread teeth. The model is validated using experimental load–displacement responses prior to thread shear failure, which is adequate for obtaining the specimen’s yield load.

### 4.3. Parametric Analysis

The effects of the key position parameters for the threaded-sleeve-type disconnectable coupling on the bearing performance of the joint were analyzed. Nineteen finite element models with different key position parameters denoted as TDC_1–19_ were established. The model parameters are listed in Table 1. The model parameters comprise the elongation (*h*_S_), nut outer diameter (*D*_S_), screw pitch (*p*_S_), screw thickness (*d*_S1_), and nut thickness (*d*_S2_). A schematic diagram of the parameters is shown in Figure 19. *F*_yS,FE_ and *k*_S,FE_ denote the finite element model yield load and initial compression stiffness, respectively.

Table 2 presents three supplementary model cases in addition to the 19 parametric-analysis models in this study. These cases adopt randomly selected key parameters and are mainly used for comparative validation of the calculation formula derived by subsequent fitting.

#### 4.3.1. Elongation

Figure 20 shows the effects of elongation on the coupling load–displacement curve. In this study, *F_yS,Ex_* denotes the yield load of the threaded coupling, and *k_S,Ex_* represents its initial compressive stiffness. The influence of elongation becomes more evident and the bearing capacity of the active end decreases as the elongation increases. The yield load and initial compression stiffness are linearly related to the elongation. For every 40 mm increase in elongation, the yield load and initial compression stiffness are reduced by approximately 300 kN and 230 kN/mm, respectively.

#### 4.3.2. Nut Outer Diameter

The threaded-sleeve-type disconnectable coupling in this study is a steel support with an outer diameter of 609 mm. The outer diameter of the nut is 610 mm. To investigate if the coupling is suitable for steel supports of different sizes, the outer diameter of the nut was varied.

The effects of the outer diameter of the nut on the bearing capacity of the threaded-sleeve-type end are shown in Figure 21a. The yield load of the joint is more significantly affected by the outer diameter of the nut. As the outer diameter increases from 610 to 1010 mm, the yield load increases by 2330 kN and the initial compression stiffness by 1456.25 kN/mm, as shown in Figure 21b. The thickness of the fan annular plate is linearly and positively correlated with the joint bearing capacity. For every 100 mm increase in the outer diameter of the nut, the yield load and initial compression stiffness increase by an average of 582.5 kN and 364.1 kN/mm, respectively.

#### 4.3.3. Screw Pitch

Figure 22a presents the influence of screw pitch on the mechanical behavior of the threaded-sleeve-type disconnectable coupling. Compared with other key geometric parameters, the variation in screw pitch produces a relatively limited influence on the bearing performance. As the screw pitch increases, both the joint yield load and initial compressive stiffness decrease approximately linearly, as plotted in Figure 21b. For every 2 mm increment in screw pitch, the yield load decreases by an average of 89 kN and the initial compressive stiffness reduces by an average of 9.4 kN/mm.

#### 4.3.4. Nut Thickness

Figure 23 illustrates the effect of nut thickness on the load–displacement response of the threaded-sleeve-type disconnectable coupling. Nut thickness exerts a remarkable influence on the mechanical performance of the coupling. When the nut thickness is no less than 20 mm, both the yield load and initial compressive stiffness increase with growing nut thickness, showing an approximate positive-correlation trend. For every 5 mm increment in nut thickness, the yield load rises by about 240 kN and the initial compressive stiffness increases by roughly 540 kN/mm. In contrast, when the nut thickness is below 20 mm, the failure mode of the joint changes, leading to a sharp drop in yield load.

#### 4.3.5. Screw Part Thickness

Figure 24 shows the effects of the screw thickness on the load–displacement curve of the threaded-sleeve-type disconnectable coupling. The screw thickness has a more significant effect and is linearly and positively correlated with the bearing performance of the active end when the thickness is less than 30 mm. For every 5 mm increase in screw thickness, the yield load increases by approximately 1510 kN and the initial compression stiffness decreases by approximately 240 kN/mm. When the screw thickness is increased to 30 mm, the change in the failure mode of the specimen results in an increased yield load and decreased the initial compression stiffness.

## 5. Fitting Formulae

Using the established finite element model, the parameters of the threaded-sleeve-type disconnectable coupling were studied, and the effects of the loading mode, geometric parameters, and material parameters on its bearing capacity and bending resistance were analyzed. The parameter analysis results were fitted to obtain formulae for the yield load and initial compression stiffness of the coupling under an axial load.

### 5.1. Calculation Formula for Yield Load

The test and numerical analysis results indicate that the main yield mechanism of the threaded-sleeve-type disconnectable coupling comprises the compression yield of the extended part of the screw part, shear failure of the thread, and compression yield of the fixed part. The yield load is the minimum value among these three contributions, which are denoted as *F*_yS,h_, *F*_yS,p_, and *F*_yS,g_. The corresponding calculation formulae for these contributions are derived in the following.

The yield load of the extended part of the screw, *F_y_*_S,h_, is given by

(1) $$F_{\mathrm{yS},h}=f_{\mathrm{yS}}\pi \left(\frac{d1^{2}-d0^{2}}{4}\right)$$

where *f*_yS_ is the yield load of the screw material, *d*_0_ the inner diameter of the screw part, and *d*_1_ the small diameter of the screw thread. *d*_1_ is given by

(2) $$d1=d_{S2}+d_{0}$$

where *d*_s2_ is the thickness of the screw part.

The yield load, *F*_yS,p_, at the time of thread shear failure is given by

(3) $$F_{\mathrm{yS},p}=z_{\mathrm{ef}}\pi d_{1}b_{S}\tau$$

where *z*_ef_ is the number of effective bearing threads. For the screw-type active end, *z*_ef_ is a parameter dependent on *H*_S_ and the total number of threads, *z*. The total number of threads, *z*, is given by

(4) $$z=\frac{H_{s}}{p_{s}}$$

A larger value of *z* results in a better bearing performance of the screw, which depends mainly on *z*_ef_. There is currently no exact formula for *z*_ef_. Yang [32] reported that more than 85% of the load is borne by the first seven turns of a thread bore. Therefore, in this study, *z*_ef_ was defined based on Yang’s findings as

(5) $$z_{\mathrm{ef}}=\left\{\begin{array}{ll} 7 & \left(z>7\right)\\ z & \left(z\leq 7\right) \end{array}\right.$$

In addition, *b*_S_ = 0.75 *p*_S_ is the width of the threaded tooth base, as shown in Figure 25; *τ* is the shear strength of the thread material and is given by *τ* = 0.6*f_yS_*.

The yield load of the fixed part, *F*_yS,g_, is given by

(6) $$F_{\mathrm{yS},g}=f_{\mathrm{yg}}\pi (D_{s}d_{s1}-{d_{s1}}^{2})$$

where *f*_yg_ is the yield strength of the fixed part material, *D*_s_ the outer diameter of the nut and fixed part, and *d*_s1_ its thickness.

Although *h_S_* and *d*_S_ do not affect the bearing capacity of the joint in the structural analysis results, the numerical results indicate that *h*_S_ and *d*_S_ affect the bearing capacity of the screw-type active end. The yield load influence coefficient, *λ*_S_, related to *h*_S_ and *d*_S_ was, therefore, obtained as

(7) $$\lambda_{S}=1.207{h_{S}}^{-0.02}$$

by fitting the numerical results. The formula for the yield load of the threaded-sleeve-type active end *F*_yS,Eq_ is, thus, obtained as

(8) $$F_{\mathrm{yS},\mathrm{Eq}}=\lambda_{S}\cdot \min\{F_{\mathrm{yS},h},F_{\mathrm{yS},p},F_{\mathrm{yS},g}\}$$

### 5.2. Calculation Formula for Initial Compression Stiffness

The compression stiffness is an important index for evaluating the deformation capacity of threaded-sleeve-type disconnectable couplings, especially in the elastic stage. The test and numerical analysis results indicate that the deformation of the joint originated mainly from the compression of the screw extension part, shear deformation of the screw thread, and compression of the fixed part. The initial compression stiffness of the coupling can, thus, be obtained by calculating the stiffness values of the three parts.

The compression stiffness of the extended part of the screw, *k*_Sh_, is given by

(9) $$k_{\mathrm{Sh}}=\frac{E_{S}\pi \left({d_{1}}^{2}-{d_{0}}^{2}\right)}{4hs}$$

where *E*_S_ is the elastic modulus of the screw material.

The shear stiffness of the thread, *k*_Sp_, is given by

(10) $$k_{\mathrm{Sp}}=\frac{G_{S}z_{\mathrm{ef}}\pi d_{1}b_{S}}{H_{S}}$$

where *G_S_* is the shear modulus of the screw material and is given by $G_{S}=\frac{E_{S}}{2(1+\nu )}$.

The compression stiffness of the fixed part, *k_Sg_*, is given by

(11) $$k_{\mathrm{Sg}}=\frac{E_{g}\pi (D_{s}d_{s1}-{d_{s1}}^{2})}{h_{g}}$$

Therefore, the initial compression stiffness (*k*_S,Eq_) of the threaded-sleeve-type disconnectable coupling can be calculated from *k*_Sh_, *k*_Sp_, and *k*_Sg_ as

(12) $$\frac{1}{k_{\mathrm{yS},\mathrm{Eq}}}=\mu_{S}\left(\frac{1}{k_{\mathrm{Sh}}}+\frac{1}{k_{\mathrm{Sp}}}+\frac{1}{k_{\mathrm{Sg}}}\right)$$

where *μ_S_* is the influence coefficient of the coupling initial compression stiffness. Regression analysis was performed on the numerical results to obtain the fitting formula:

(13) $$\mu_{S}=0.028D_{S}^{0.43}p_{s}^{0.67}$$

### 5.3. Comparison Between Calculation and Numerical Results

The yield load and initial compression stiffness values of the threaded-sleeve-type disconnectable coupling obtained from the numerical simulation and calculation formulae are listed in Table 3 along with the ratios of the numerical simulation results to the calculation results. Including No. 20~No. 22 of random FEMs, the yield load ratios of more than 80% of the joints and initial compression stiffness ratios of nearly 90% of the joints fall between 0.9 and 1.1. This demonstrates that the established fitting formulae can calculate the yield load and initial compression stiffness values of disconnectable couplings with acceptable accuracy.

### 5.4. Comparison Between Calculation and Test Results

Table 4 lists the yield load and initial compression stiffness values obtained from the threaded-sleeve-type disconnectable coupling test and the fitting formulae along with their ratios. The calculation results are in good agreement with the test results. The differences in the yield loads are less than 5% and those in the initial compression stiffness less than 10%. This demonstrates that the established calculation formulae for the end yield load and initial compression stiffness of the coupling can be used to predict its bearing capacity in engineering applications. The formulae provide a theoretical basis for the design and analysis of threaded-sleeve-type disconnectable couplings.

## 6. Discussion

It should be emphasized that both the full-scale test and numerical analyses in this study are performed under ideal pure axial compression. In practical foundation pit engineering, the coupling may encounter eccentric compression, installation misalignment, local bearing, preload loss, construction disturbance and cyclic load variations. The conclusions drawn in this paper are only applicable to axial-compression conditions. The mechanical responses under eccentric, combined axial-bending and cyclic loading are not covered herein, which deserve dedicated research in future work.

## 7. Conclusions

The bearing characteristics of a threaded-sleeve-type disconnectable coupling were studied experimentally and numerically. Parametric analysis was performed, and calculation formulae for the yield load and initial compression stiffness were obtained via further fitting. The main conclusions are as follows:

1. Under an axial load, the yield load of the coupling is 10,509.96 kN, the initial compression stiffness is 4215.17 kN·mm^−1^, and the ultimate load is 12,980.2 kN. The failure mode of the coupling is the bulging deformation of the nut part and shear failure at the thread. The thread shear failure is most obvious at the first five rings.
2. The load–displacement curve shows that the coupling has good ductility. The specimen curve has an evident yield plateau. The load–strain curve shows that strain concentration in the specimen occurred at the screw and that plastic strain first occurred at the screw. Therefore, the size and material of the screw and fixed parts can be optimized to better match their strain variations to improve the full bearing performance of the coupling.
3. The bearing capacity of the coupling under an axial load is linearly and positively correlated with the outer diameter of the nut. In contrast, the bearing capacity is linearly and inversely correlated with the elongation and screw pitch bearing. The nut thickness is positively correlated with the bearing capacity of the threaded-sleeve-type active end. This correlation increases gradually with the nut thickness. The screw thickness is positively correlated with the bearing capacity of the active winding end. However, this correlation decreases gradually with an increasing screw thickness.
4. The yield load and initial compression stiffness results obtained using the calculation formulae are in good agreement with the numerical and experimental results with errors of less than 10%. The established calculation formulae can accurately estimate the yield load and initial compression stiffness values of threaded-sleeve-type disconnectable couplings. The proposed calculation methods for the axial-compressive yield load and initial compressive stiffness of the joint lay a certain theoretical foundation for its subsequent application and promotion.

## Figures and Tables

**Figure 1:**
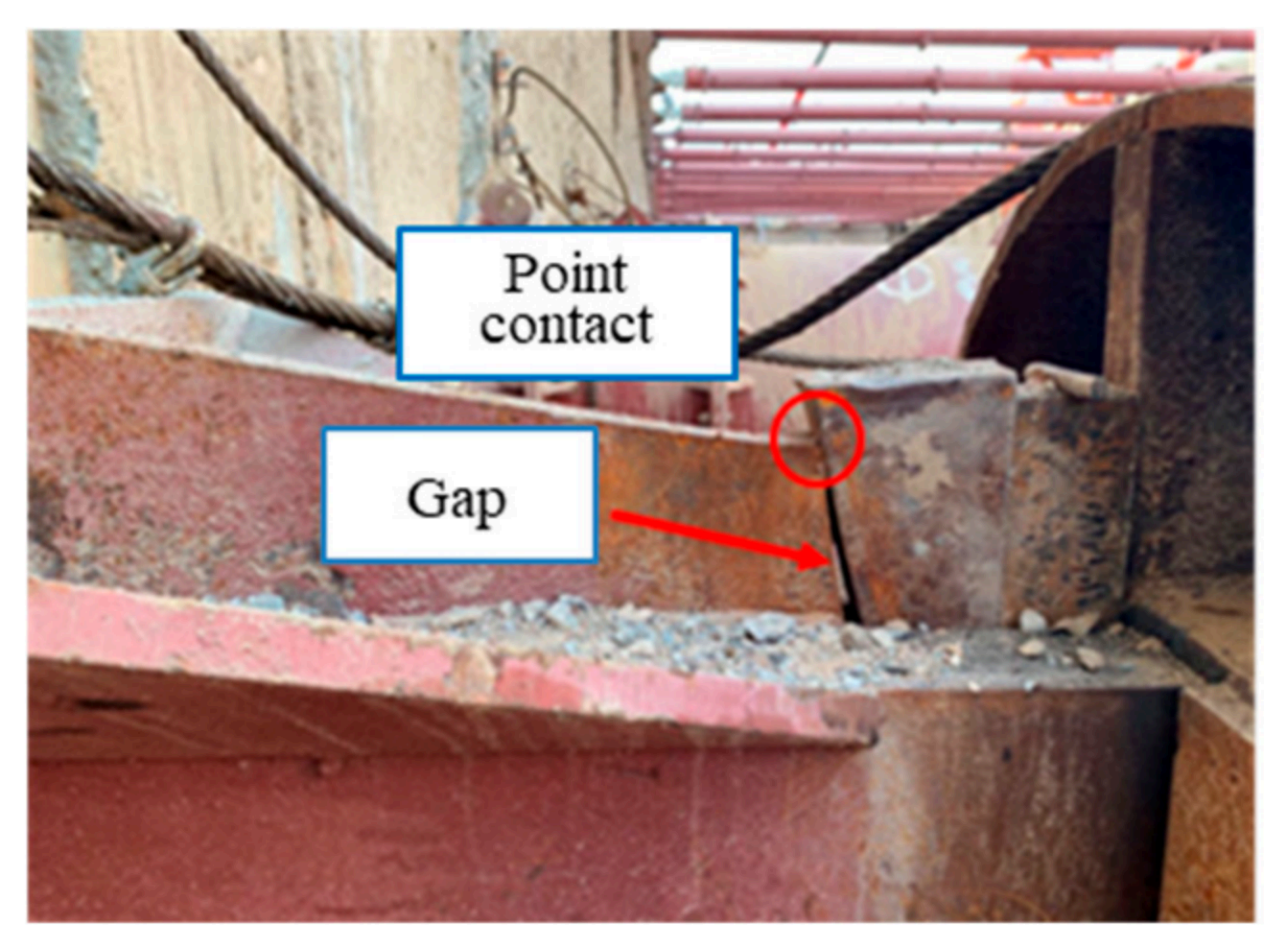
Steel wedge stress concentration.

**Figure 2:**
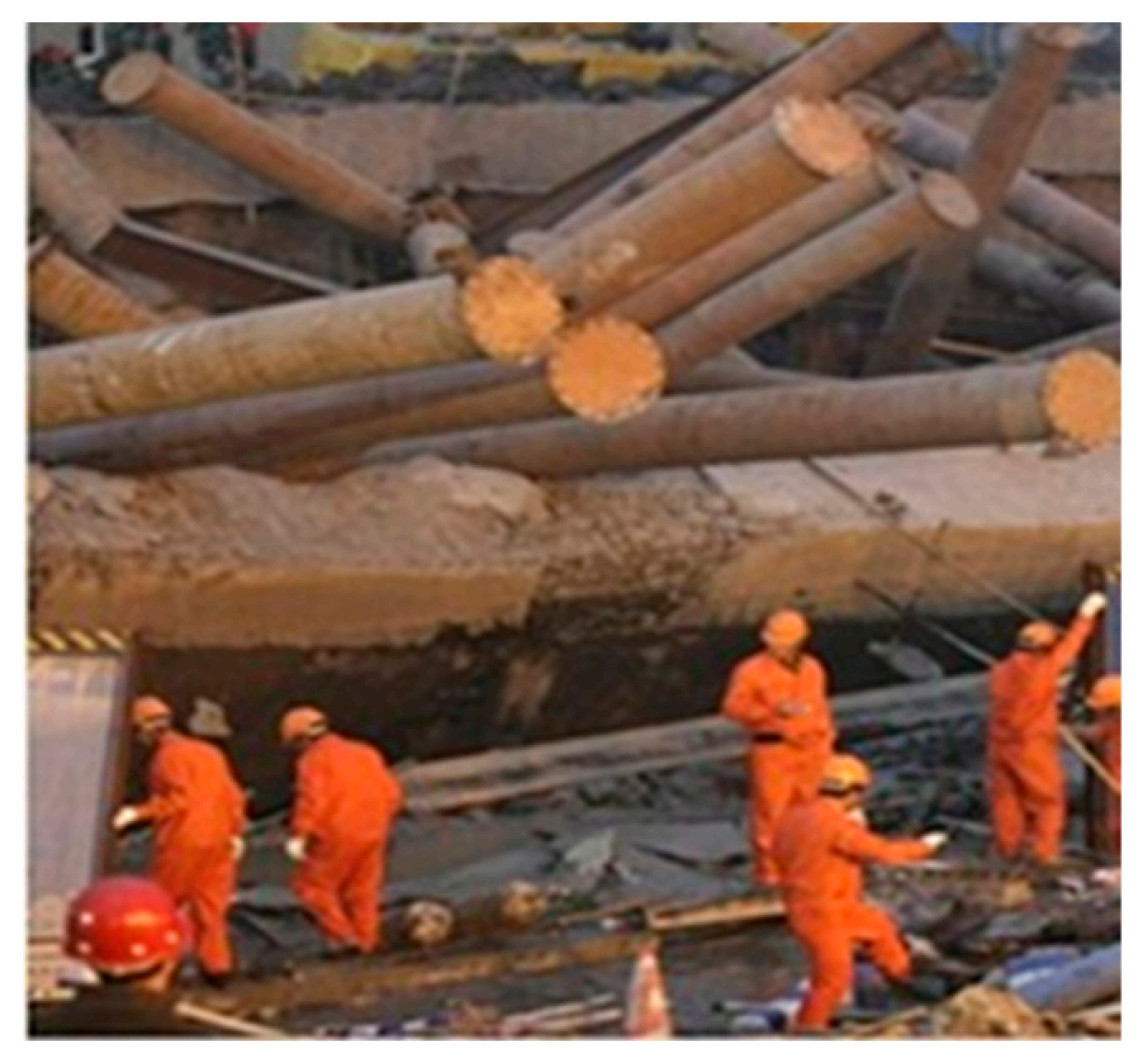
Foundation pit accident at Xianghu Station.

**Figure 3:**
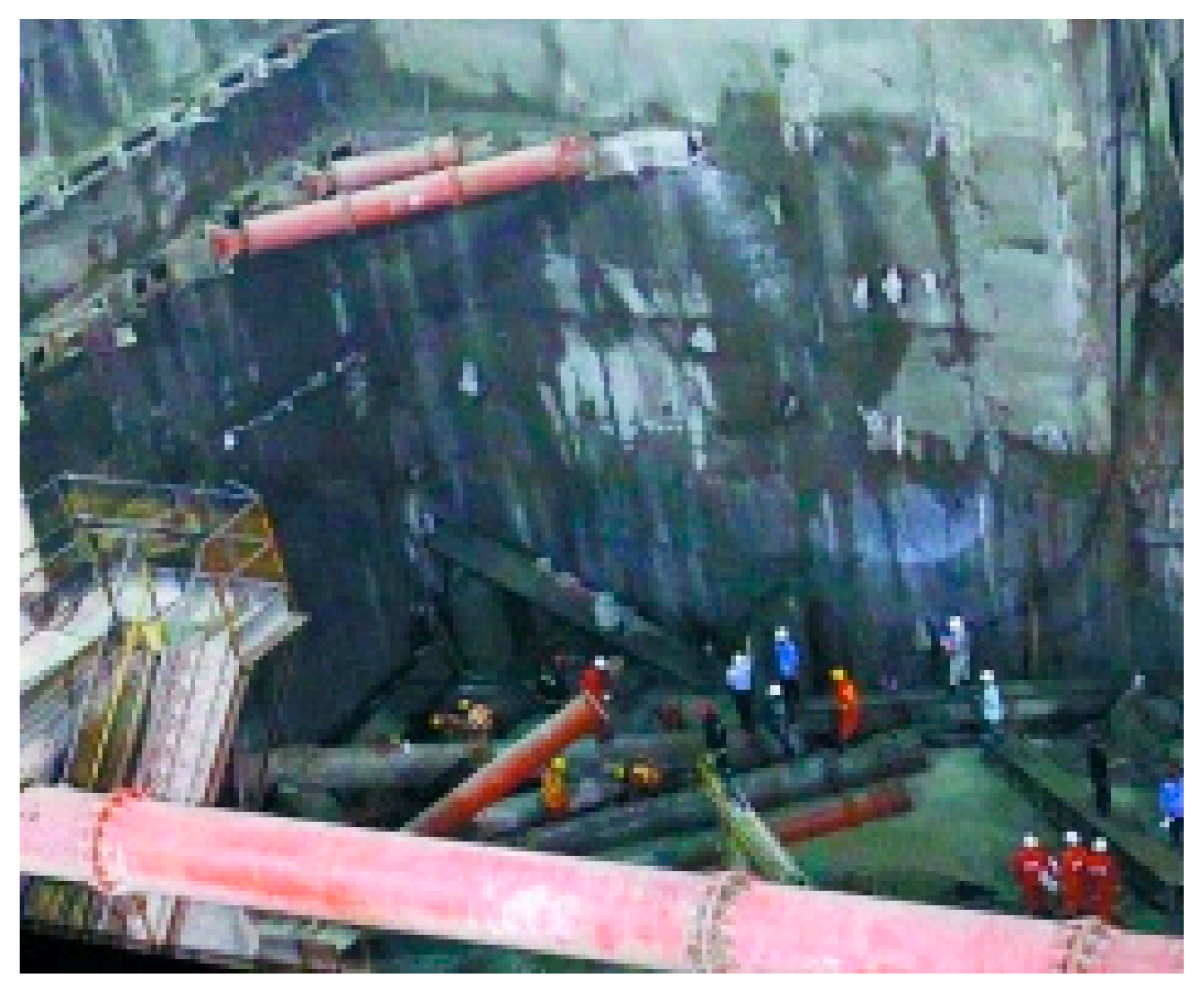
Foundation pit accident at Shunyi Station.

**Figure 4:**
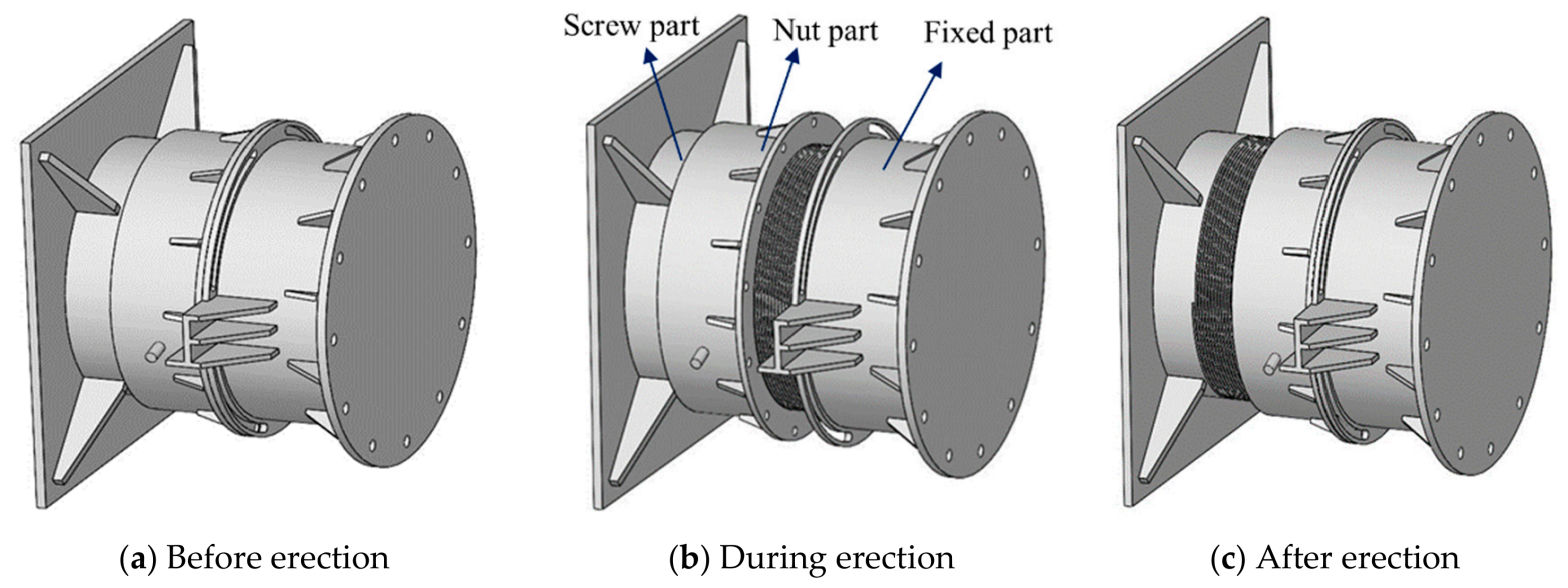
Installation of threaded-sleeve-type disconnectable coupling.

**Figure 5:**
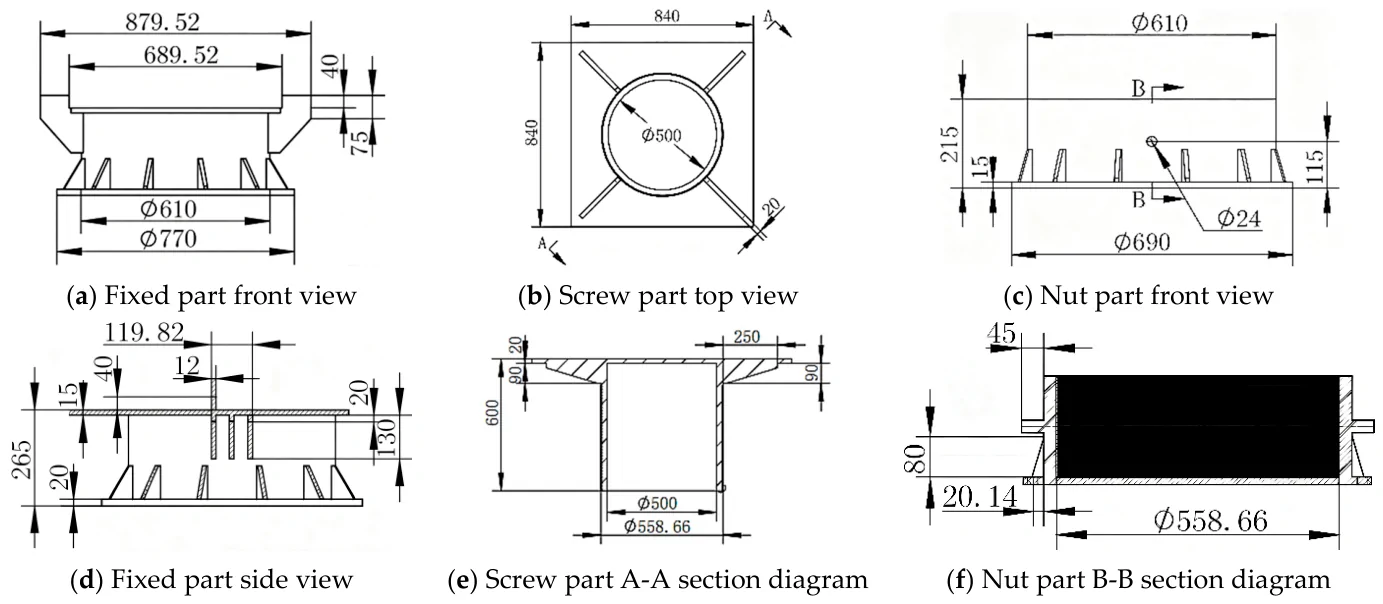
Design drawing of the threaded-sleeve-type disconnectable coupling (unit: mm).

**Figure 6:**
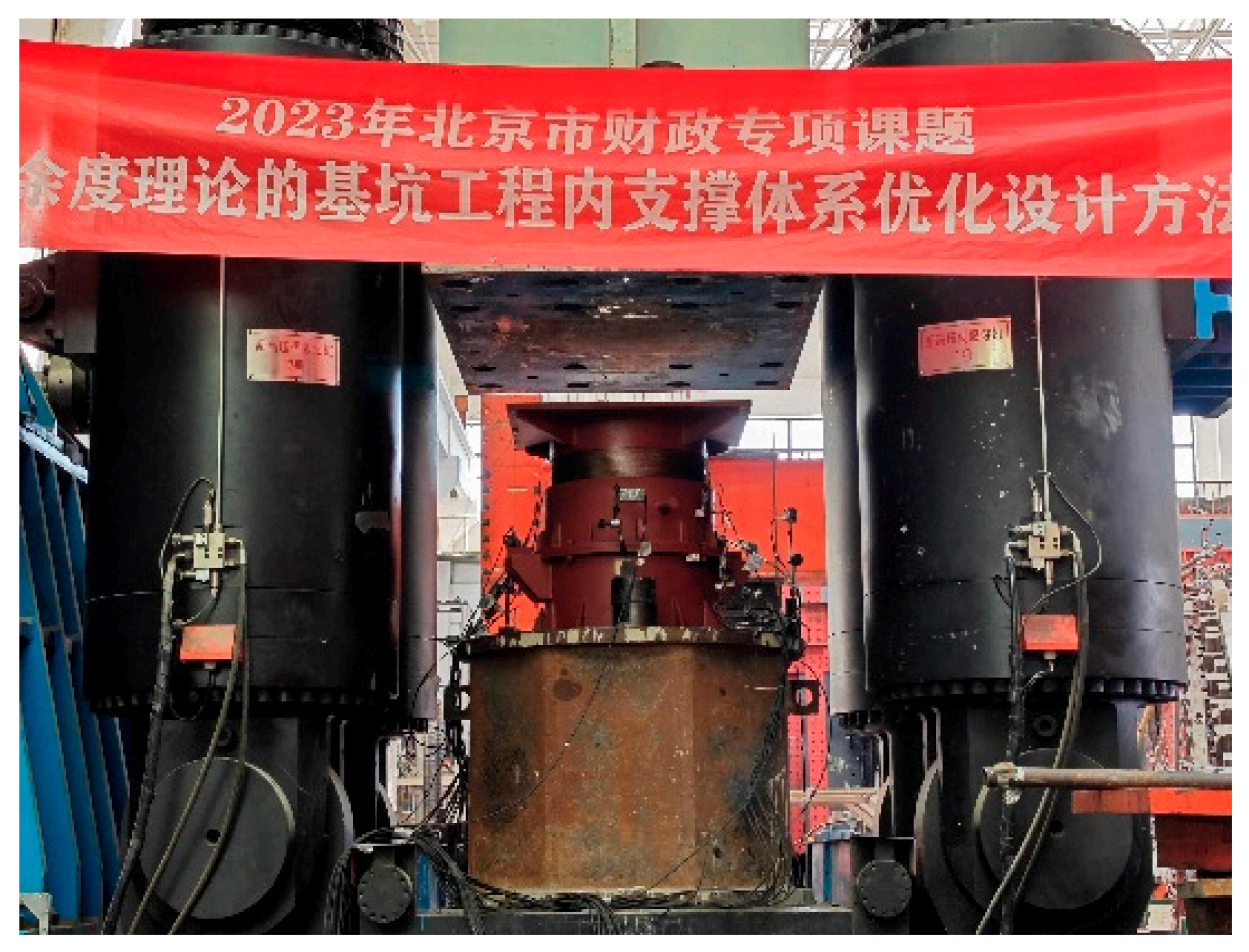
Loading device.

**Figure 7:**
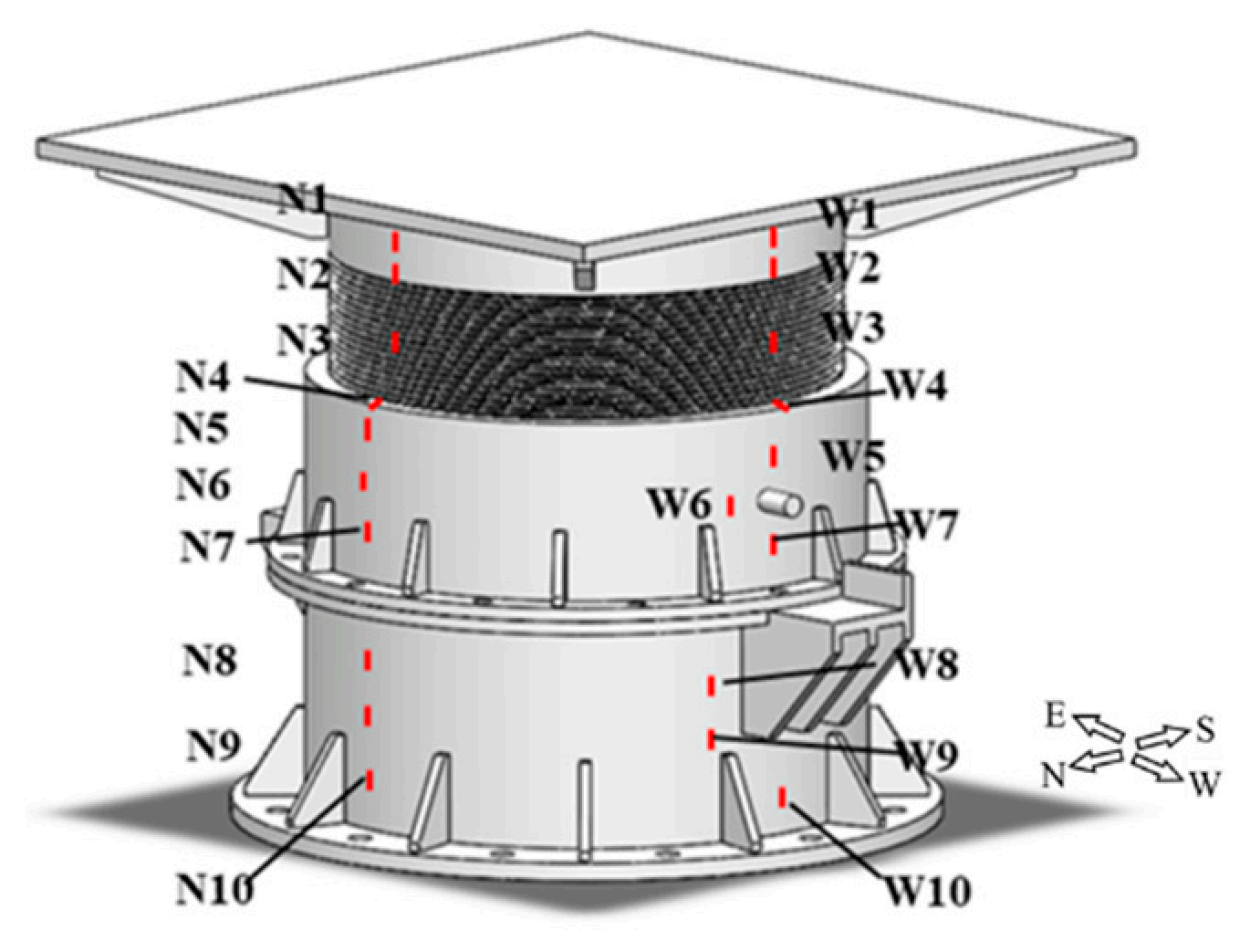
Layout of the specimen strain measurement points.

**Figure 8:**
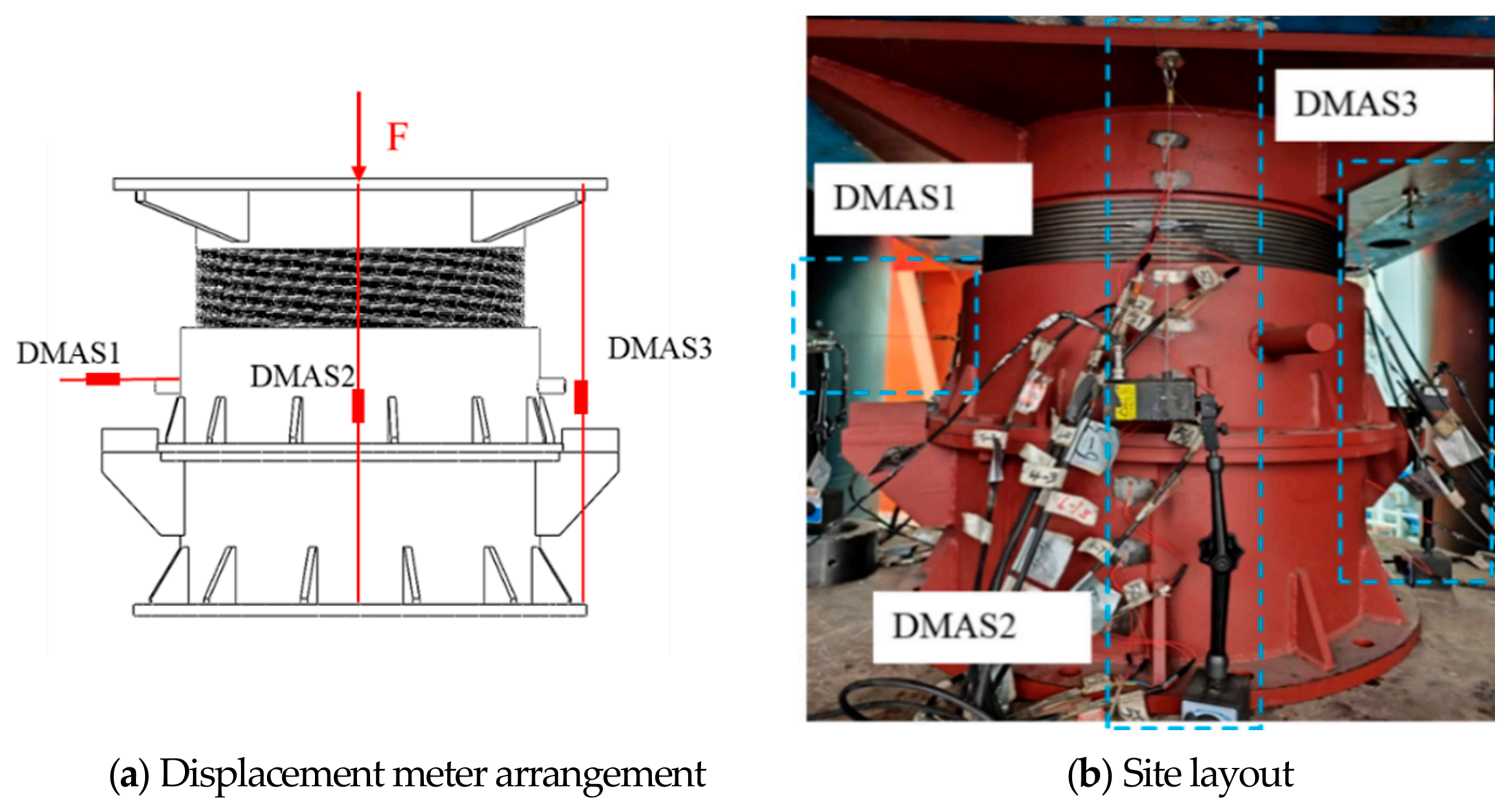
Placement of the DMAS displacement sensors on the specimen.

**Figure 9:**
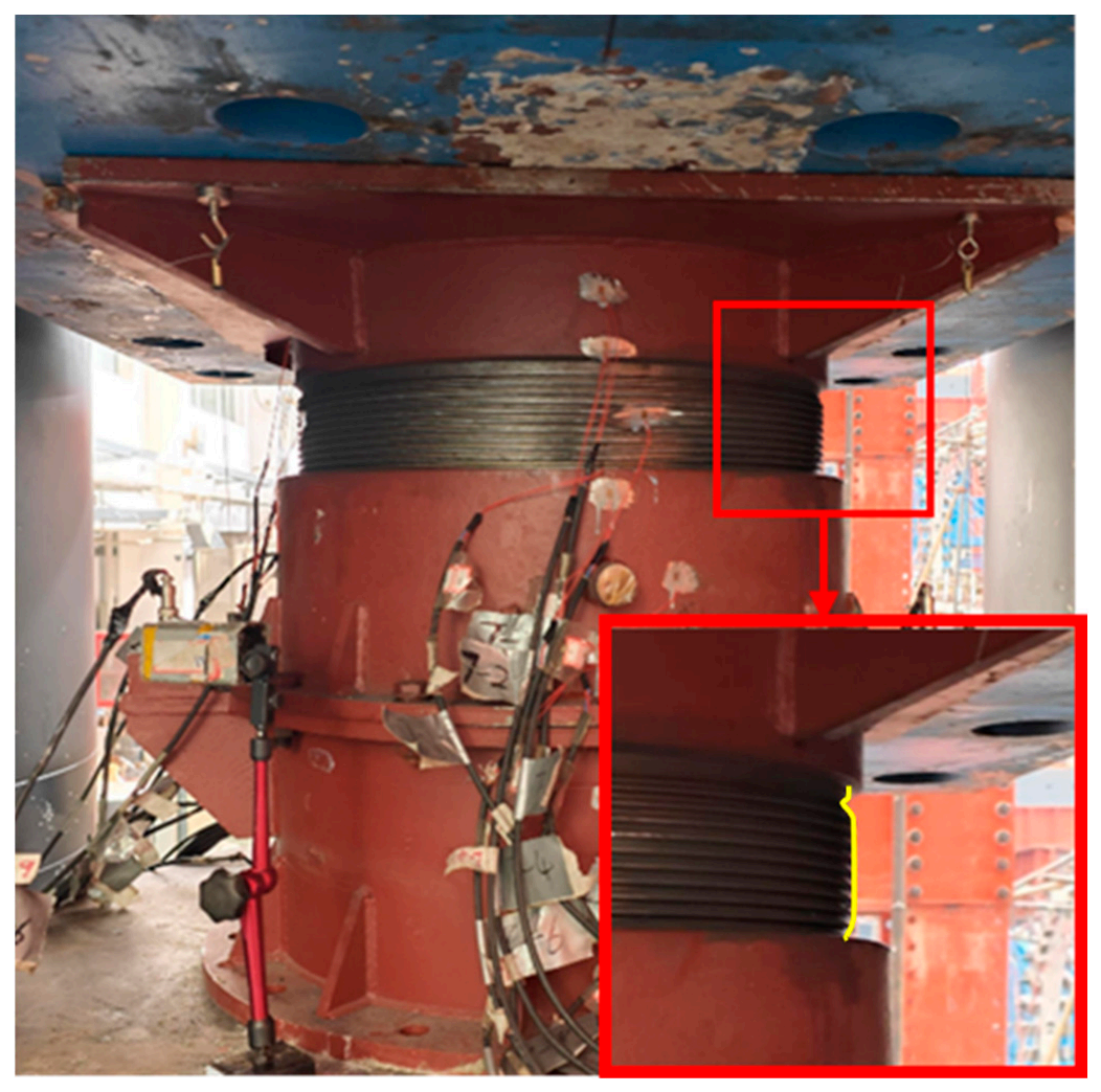
Specimen loading.

**Figure 10:**
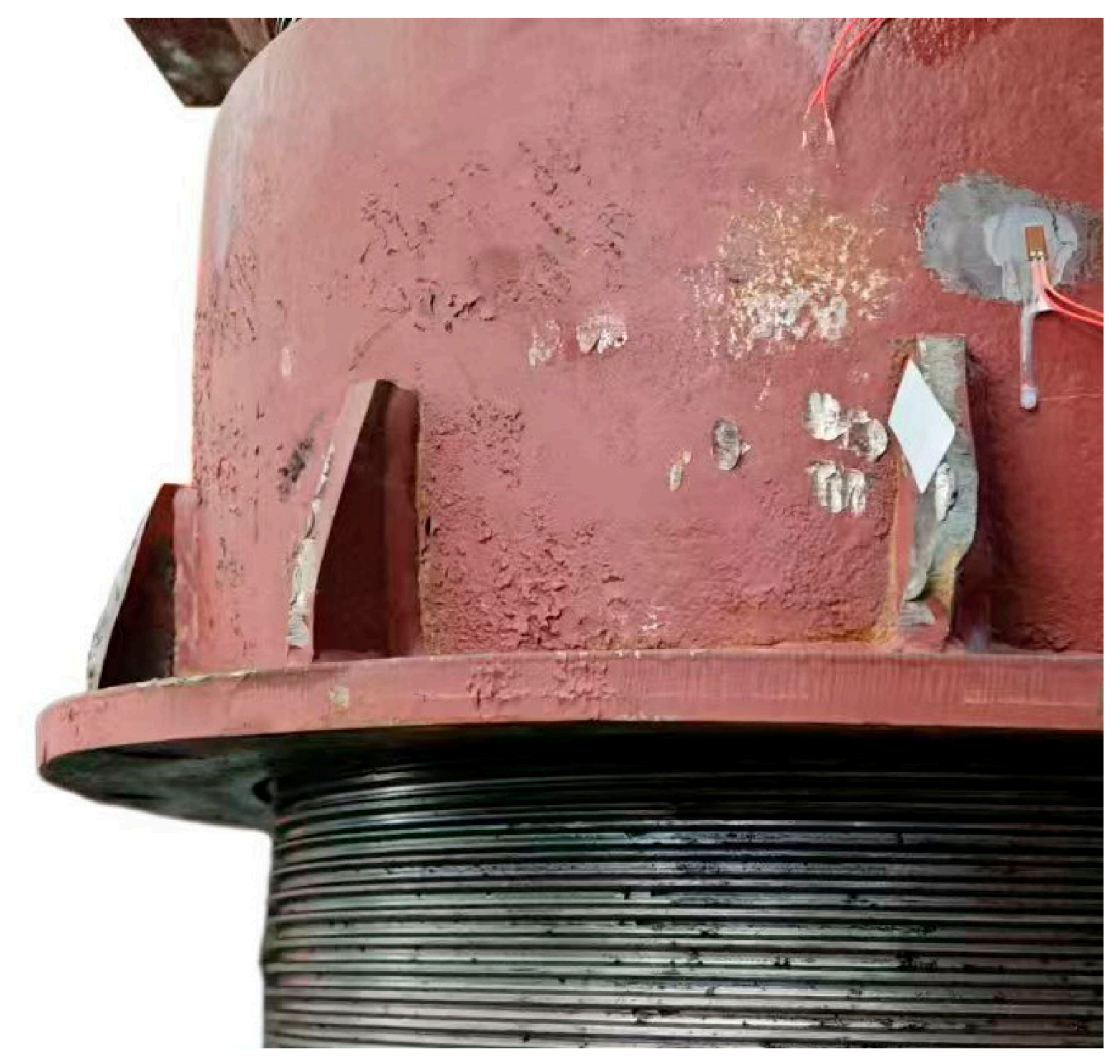
Disassembly of the nut part.

**Figure 11:**
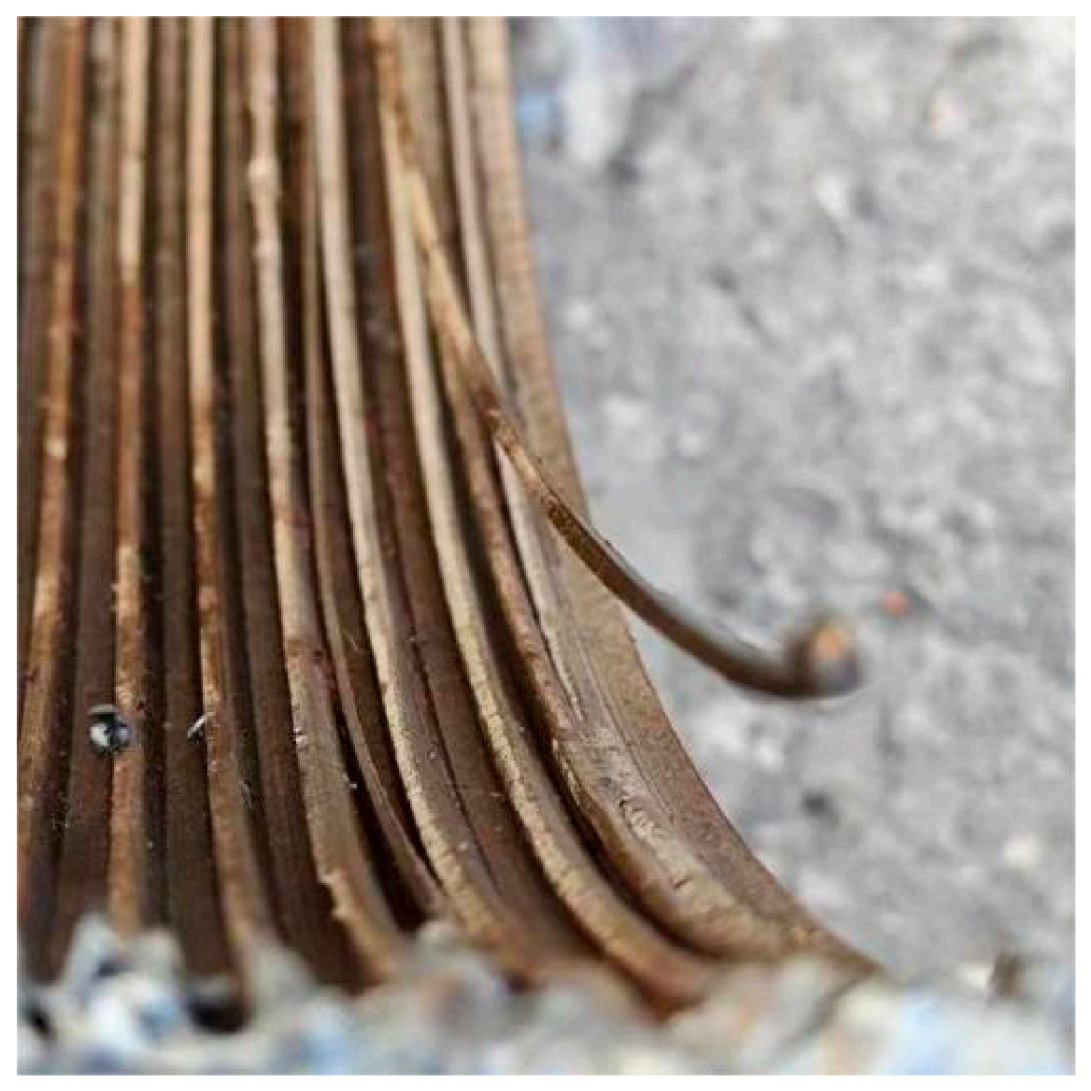
Thread shear failure mode of the threaded-sleeve-type disconnectable coupling.

**Figure 12:**
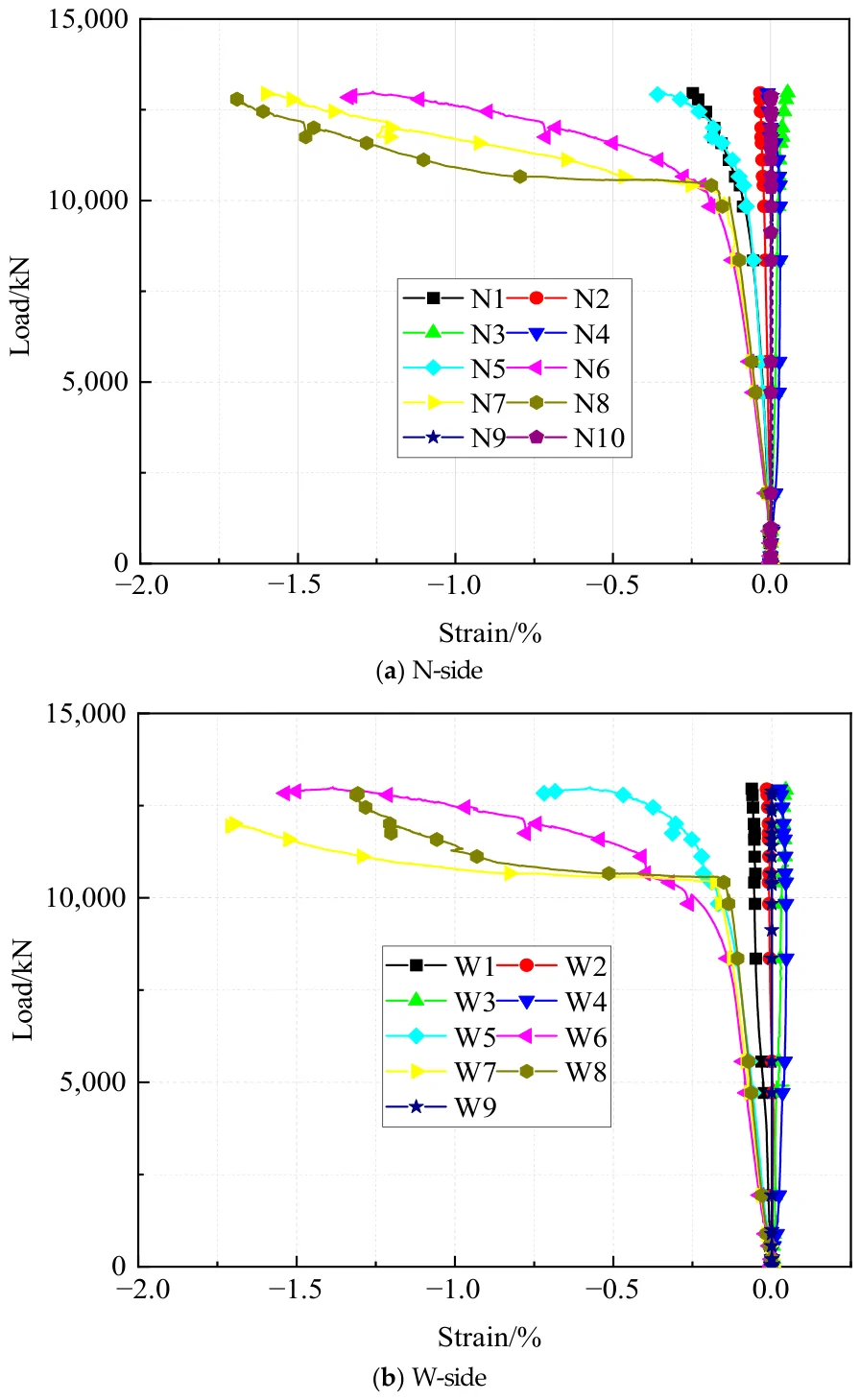
Load–strain curves.

**Figure 13:**
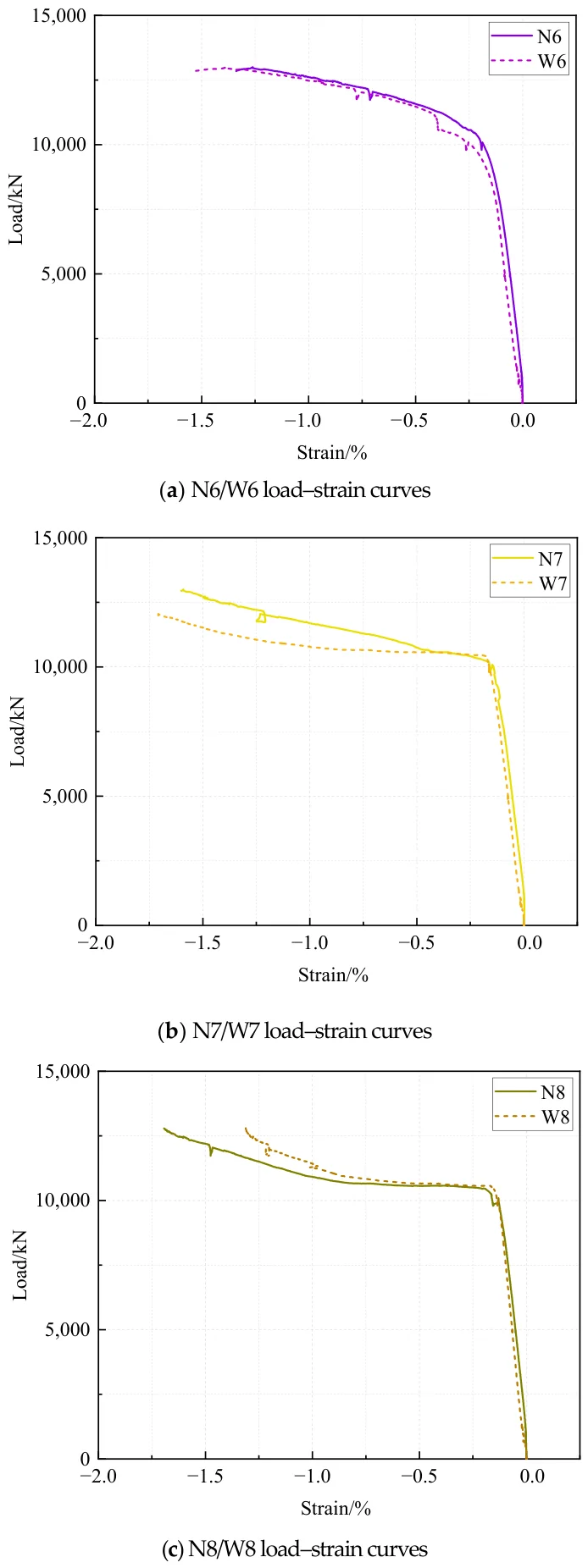
Comparison of the load–strain curves for the N-side and W-side.

**Figure 14:**
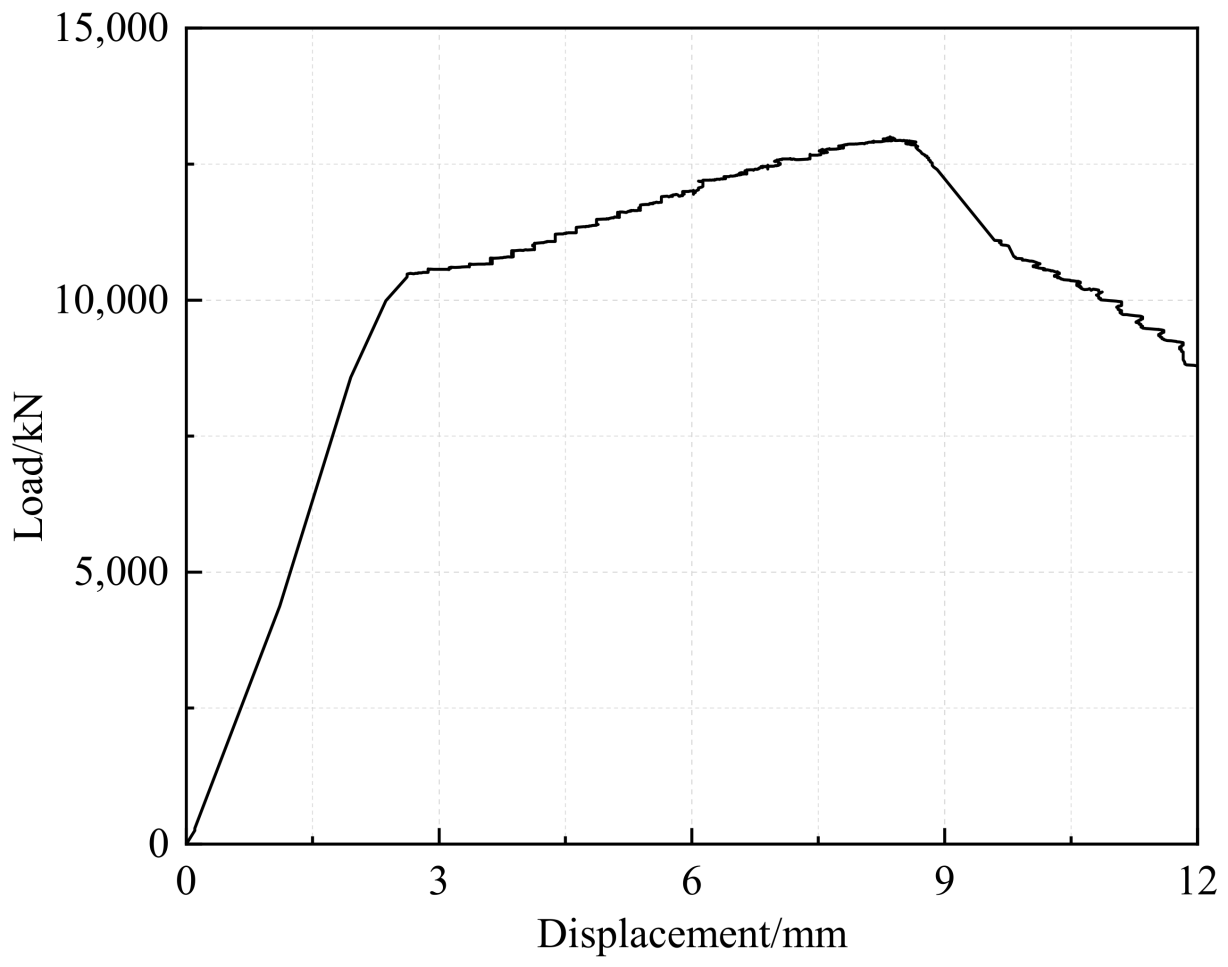
Vertical load–displacement curve.

**Figure 15:**
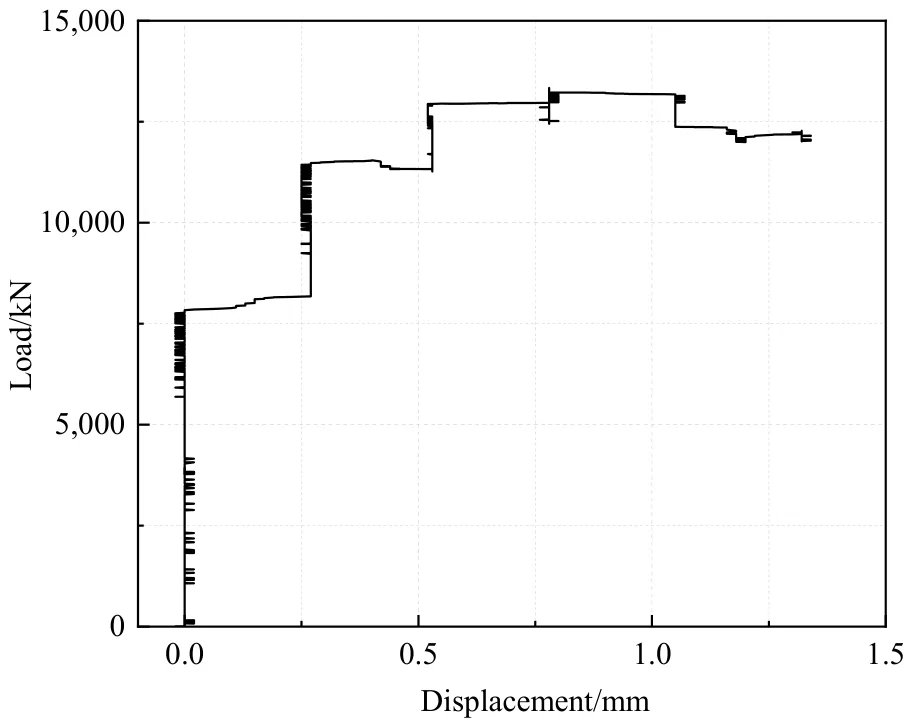
Horizontal load–displacement curve.

**Figure 16:**
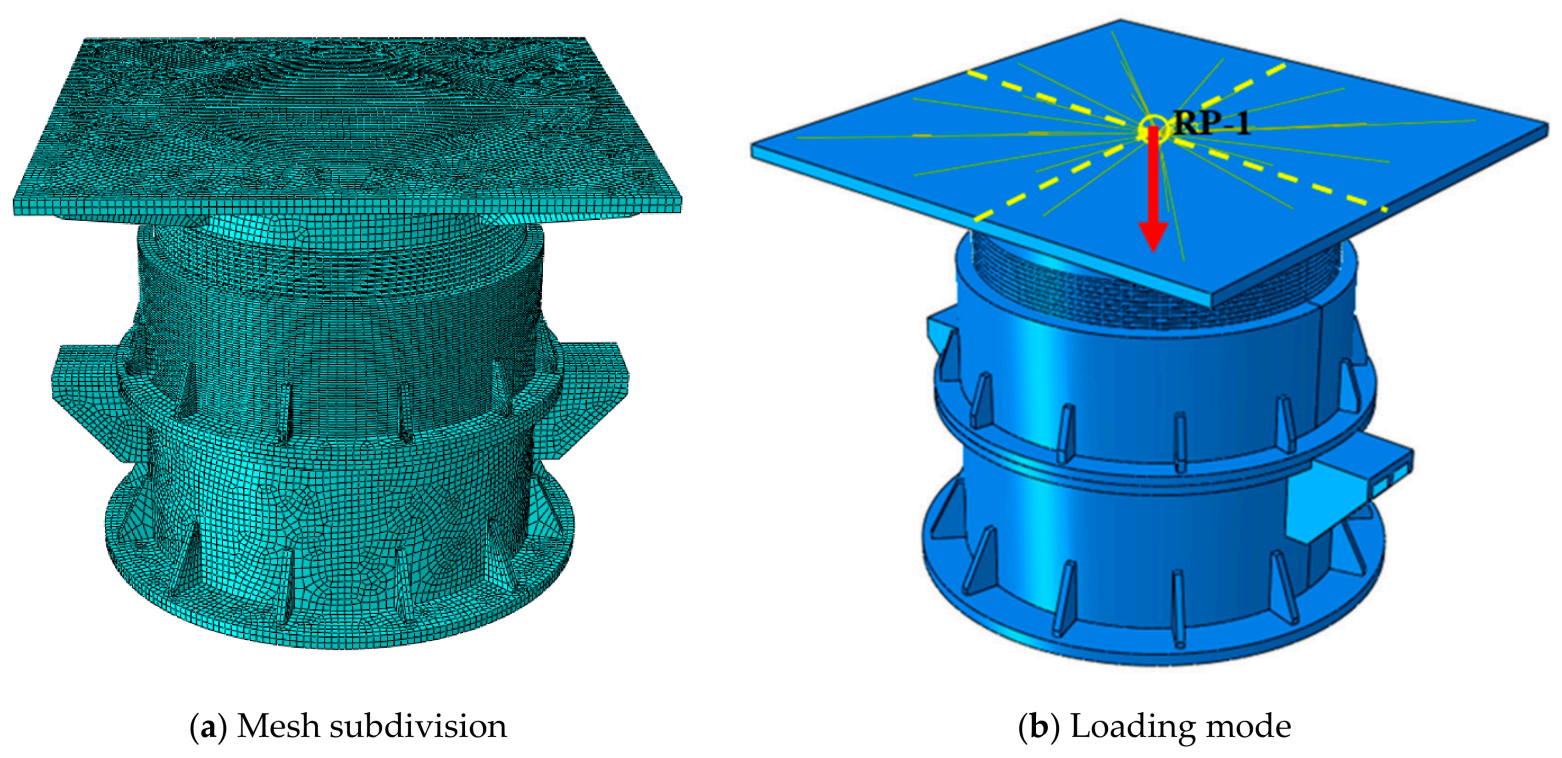
Finite element model specifications.

**Figure 17:**
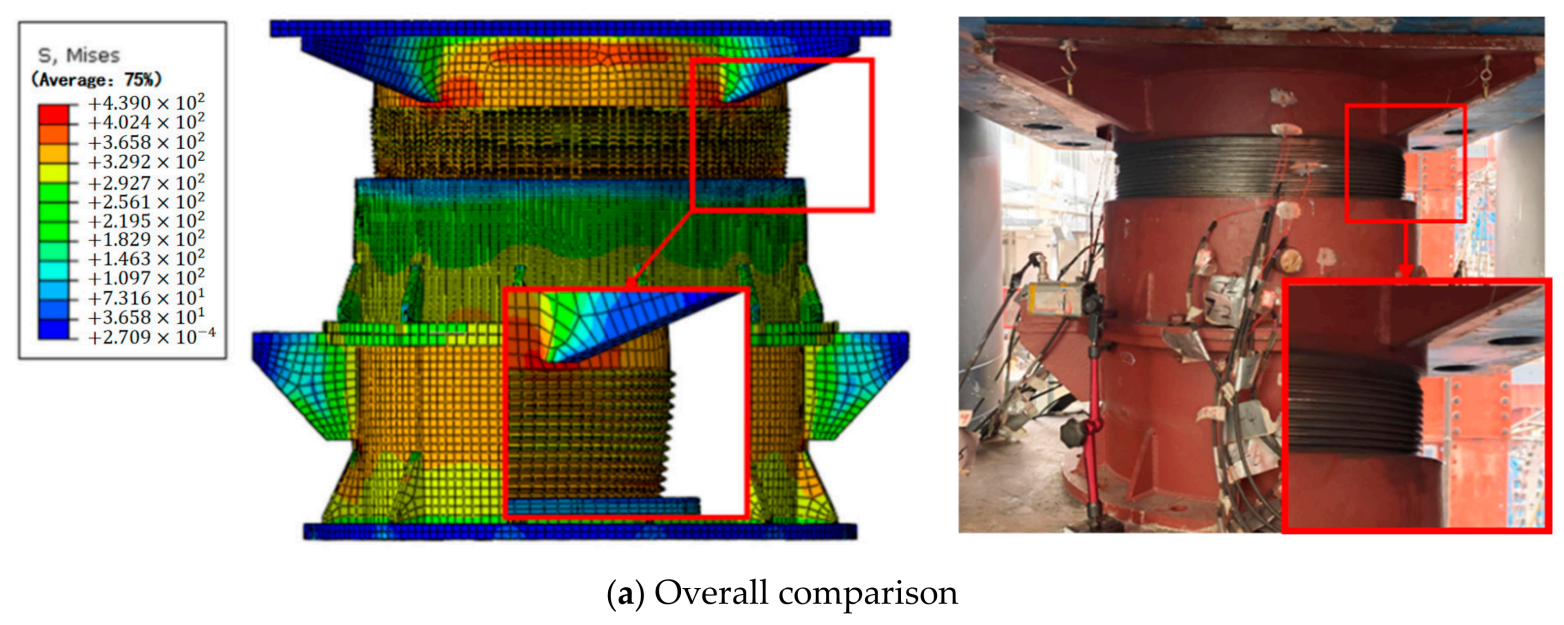

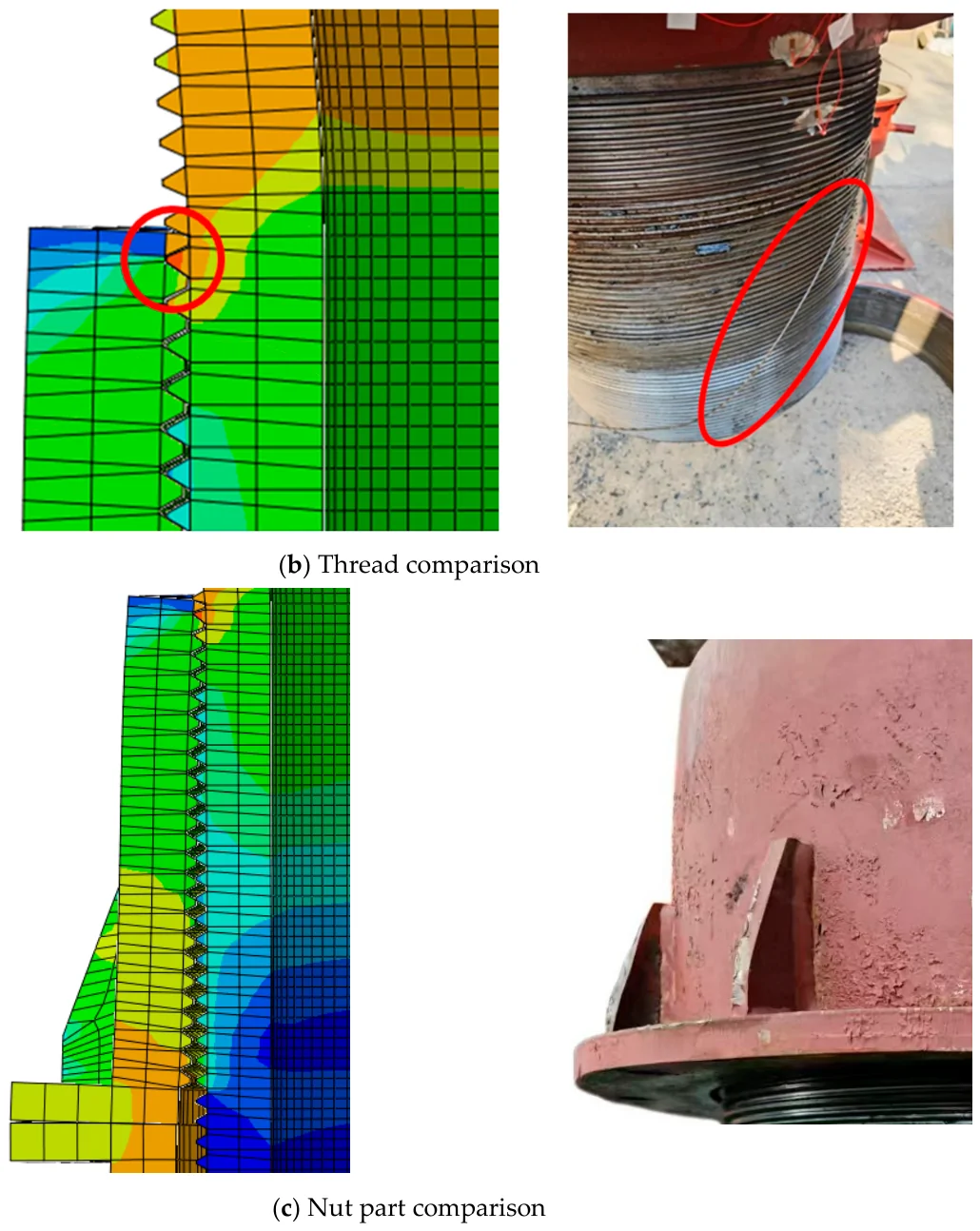
Comparison between the numerical simulation and experimental results.

**Figure 18:**
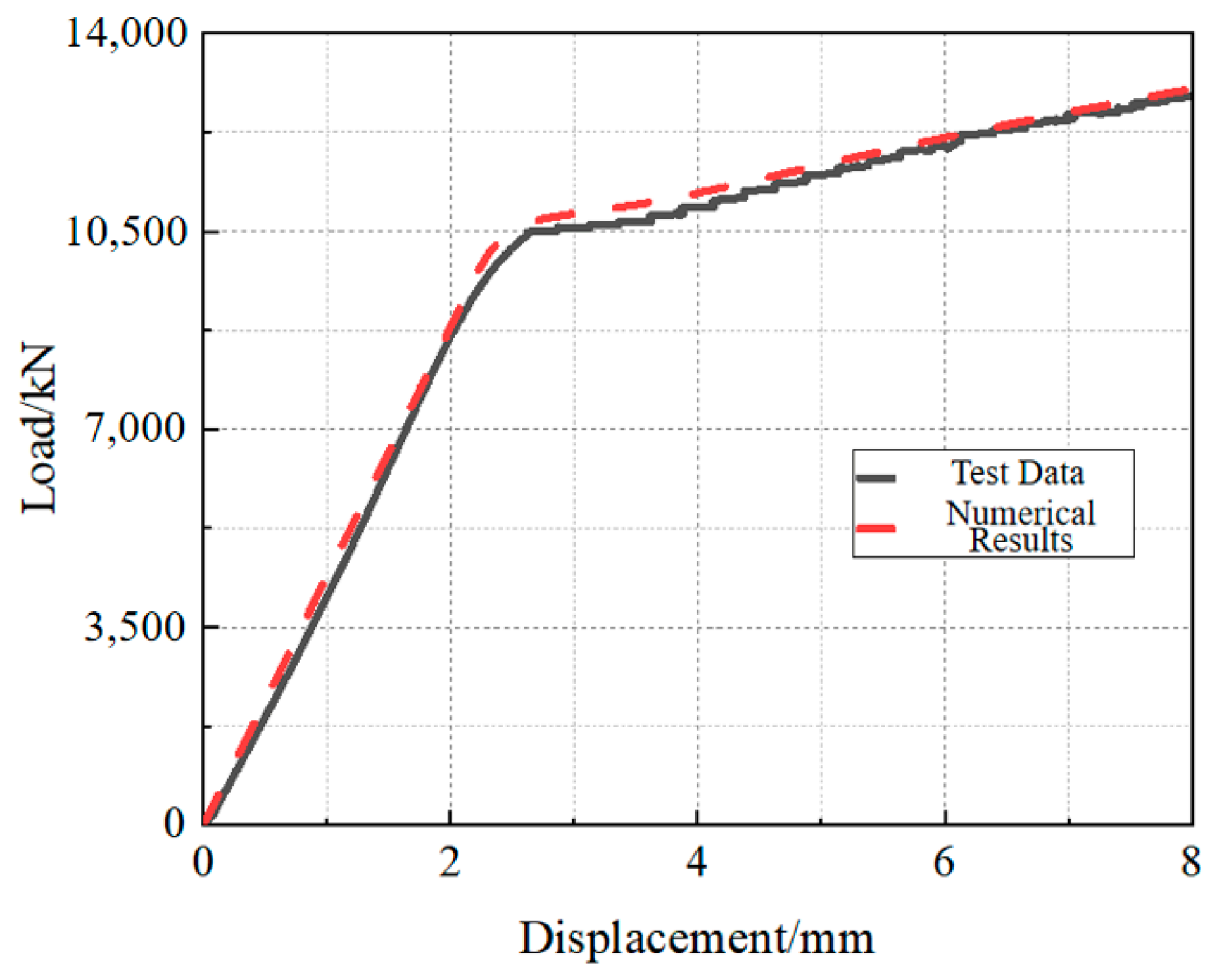
Numerical and test specimen load–displacement curves.

**Figure 19:**
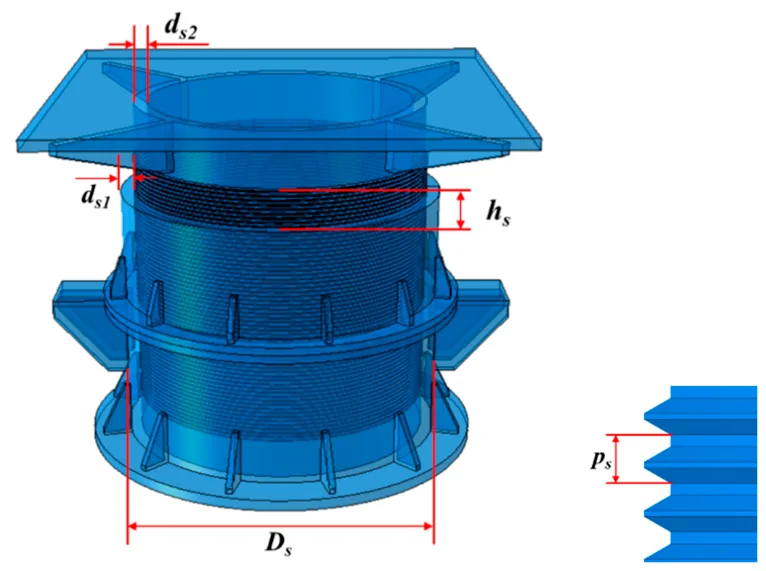
Parameters of the threaded-sleeve-type active disconnectable coupling model.

**Figure 20:**
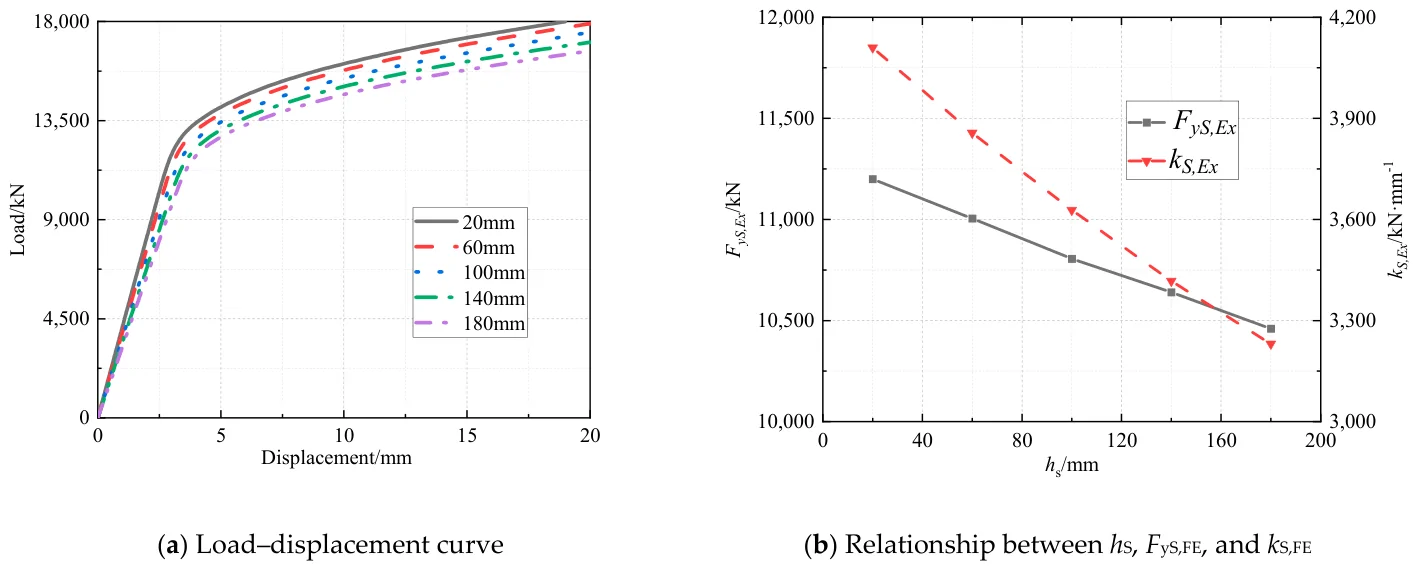
Effects of elongation on the bearing capacity.

**Figure 21:**
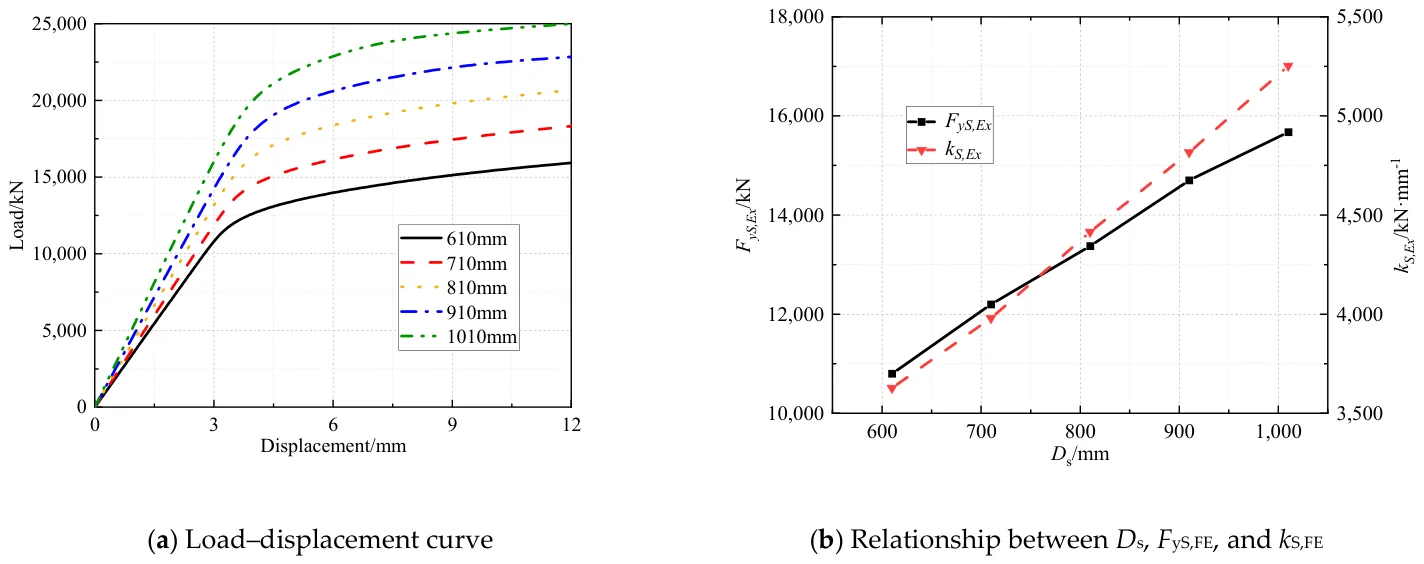
Effects of the nut outer diameter on the bearing capacity.

**Figure 22:**
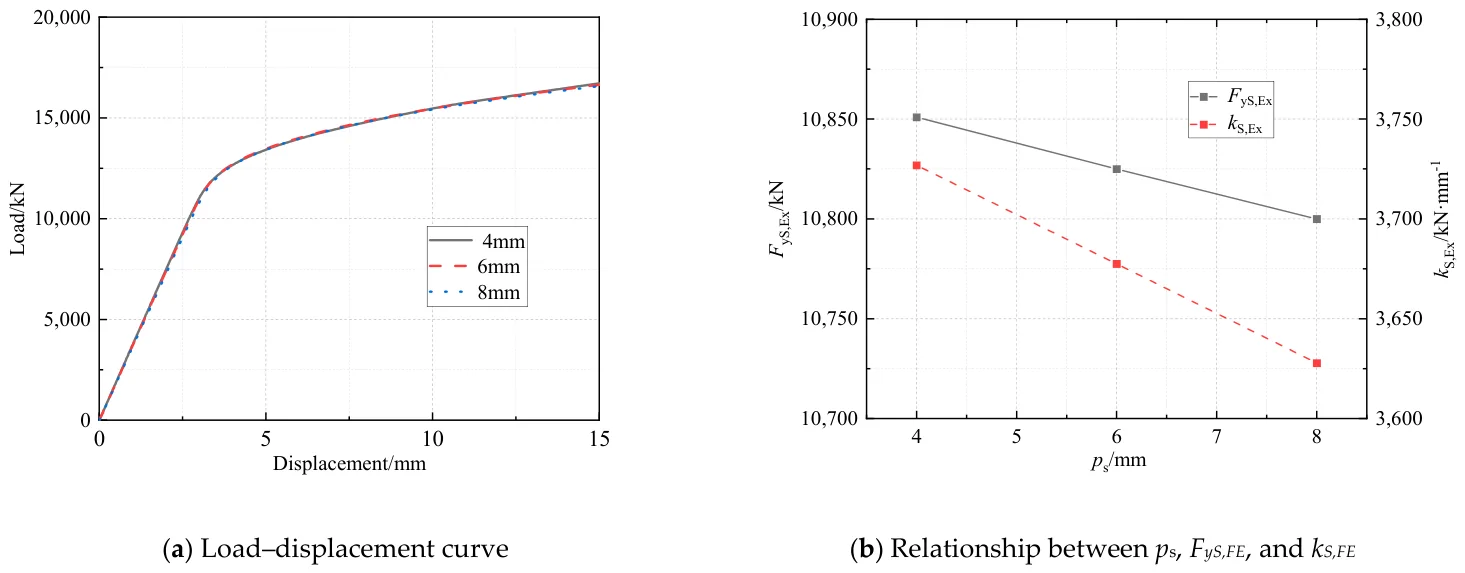
Effects of the screw pitch on the bearing capacity.

**Figure 23:**
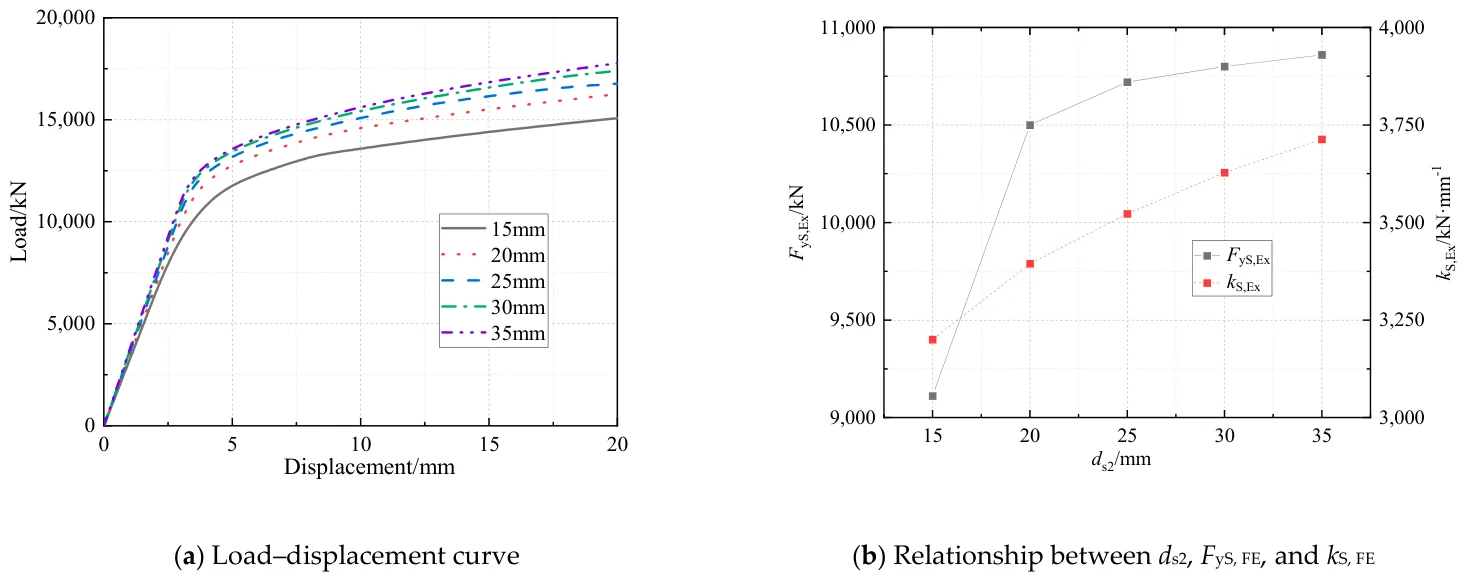
Effects of the screw pitch on the bearing capacity.

**Figure 24:**
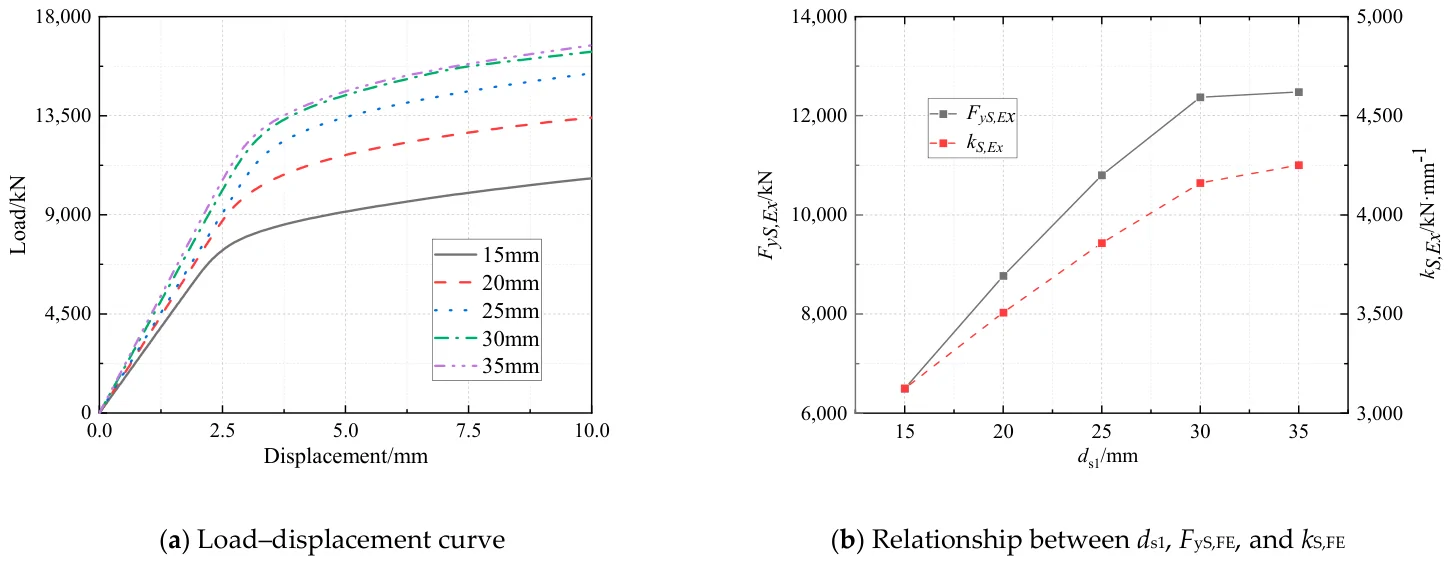
Effects of the screw part thickness on the bearing capacity.

**Figure 25:**
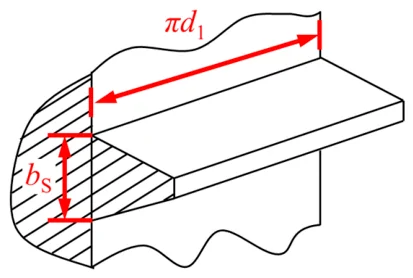
Thread expansion diagram.

**Table 1:** Key geometric position parameters of FEMs (unit: mm).

No.	*h*_S_	*D*_s_	*p*_S_	*d*_s1_	*d*_s2_
TDC_1–5_	20/60/100/140/180	610	8	30	25
TDC_3,6–9_	100	610/710/810/910/1010	8	30	25
TDC_10,3,11_	100	610	4/6/8	30	25
TDC_12,13,3,14,15_	100	610	8	15/20/25/30/35	25
TDC_16,17,3,18,19_	100	610	8	30	15/20/25/30/35

**Table 2:** Cases of stochastic parameter FEMs for validation analysis (unit: mm).

No.	*h*_S_	*D*_s_	*p*_S_	*d*_s1_	*d*_s2_
TDC_20_	120	860	8	15	35
TDC_21_	110	910	6	20	30
TDC_22_	100	610	4	25	25

**Table 3:** Comparison of numerical and calculated results.

No.	*F*_yS,FE_ (kN)	*F*_yS,Eq_ (kN)	*F*_yS,FE_/*F*_yS,Eq_	*k*_S,FE_ (kN/mm)	*k*_S,Eq_ (kN/mm)	*k*_S,FE_/*k*_S,Eq_
TDC1	11,200	11,015.49	1.02	4109.59	4761.00	0.86
TDC2	11,005	10,776.92	1.02	3857.14	4100.71	0.94
TDC3	10,806	10,658.84	1.01	3688.12	3684.50	1.00
TDC4	10,640	10,595.02	1.00	3417.41	3228.72	1.06
TDC5	10,460	10,541.90	0.99	3230.77	2926.03	1.10
TDC6	12,250	12,689.09	0.97	3980.28	4000.00	0.99
TDC7	13,745	14,719.34	0.93	4414.93	4347.83	1.02
TDC8	15,320	16,749.60	0.91	4786.38	4739.34	1.01
TDC9	17,075	18,779.86	0.91	5402.4	5076.14	1.06
TDC10	10,851	10,658.84	1.02	3726.68	3717.47	1.00
TDC11	10,825	10,658.84	1.01	3689.54	3731.34	0.99
TDC12	9011	8248.01	1.09	3216.58	3156.17	1.02
TDC13	10,500	9592.80	1.09	3397.89	3384.32	1.00
TDC14	10,740	10,658.84	1.01	3522.46	3533.32	0.99
TDC15	10,860	10,658.84	1.02	3703.28	3720.52	0.99
TDC16	6500	6517.12	0.99	3125	3053.25	1.02
TDC17	8770	8608.28	1.02	3508	3384.32	1.04
TDC18	12,374	11,255.71	1.09	4160.72	3795.93	1.09
TDC19	12,480	11,255.71	1.11	4251.42	3956.32	1.07
TDC20	20,085	21,904.05	0.92	6227.69	5938.61	1.05
TDC21	15,716	16,218.24	0.97	4987.29	5110.94	0.97
TDC22	11,192	10,617.04	1.05	3891.82	4216.57	0.92

**Table 4:** Comparison of the experimental and calculated results.

No.	*F*_yS,Ex_ (kN)	*F*_yS,Eq_ (kN)	*F*_yS,Ex_/*F*_yS,Eq_	*k*_S,Ex_ (kN/mm)	*k*_S,Eq_ (kN/mm)	*ks,*_Ex_/*k*_S,Eq_
TDC	10,509.96	10,658.84	0.99	3980.17	3684.50	1.08

## Data Availability

The original contributions presented in this study are included in the article. Further inquiries can be directed to the corresponding author.
